# From Bayes-optimal to heuristic decision-making in a two-alternative forced choice task with an information-theoretic bounded rationality model

**DOI:** 10.3389/fnins.2022.906198

**Published:** 2022-09-29

**Authors:** Cecilia Lindig-León, Nehchal Kaur, Daniel A. Braun

**Affiliations:** Faculty of Engineering, Computer Science and Psychology, Institute of Neural Information Processing, Ulm University, Ulm, Germany

**Keywords:** Bayes-optimality, heuristics, bounded rationality, decision-making, information theory

## Abstract

Bayes optimal and heuristic decision-making schemes are often considered fundamentally opposed to each other as a framework for studying human choice behavior, although recently it has been proposed that bounded rationality may provide a natural bridge between the two when varying information-processing resources. Here, we investigate a two-alternative forced choice task with varying time constraints, where subjects have to assign multi-component symbolic patterns to one of two stimulus classes. As expected, we find that subjects' response behavior becomes more imprecise with more time pressure. However, we also see that their response behavior changes qualitatively. By regressing subjects' decision weights, we find that decisions allowing for plenty of decision time rely on weighing multiple stimulus features, whereas decisions under high time pressure are made mostly based on a single feature. While the first response pattern is in line with a Bayes-optimal decision strategy, the latter could be considered as an instantiation of heuristic decision-making with cue discounting. When fitting a bounded rational decision model with multiple feature channels and varying information-processing capacity to subjects' responses, we find that the model is able to capture subjects' behavioral change. The model successfully reflects the simplicity of heuristics as well as the efficiency of optimal decision making, thus acting as a bridge between the two approaches.

## 1. Introduction

In the cognitive and neural sciences, Bayes-optimal decision-making models have advanced over the last few decades to one of the dominant formal approaches to understand reasoning and acting in humans, animals, and machines (Doya et al., 2007). These models have strong theoretical appeal due to their axiomatic simplicity and elegance that allow for a normative interpretation of how decisions should be taken optimally by rational actors (von Neumann and Morgenstern, 1944; Savage, 1954). Bayes-optimal models typically consist of two distinct components: first a model of beliefs that represent the states of unobserved variables taking into account the subjective uncertainty which is updated with new incoming data, and second an optimal decision-making scheme that selects actions in a way that maximizes the expected utility of the decision-maker given the current beliefs. Although it has been noted early on in the economic sciences that human decision-makers can substantially deviate from Bayes-optimality (Allais, 1953; Ellsberg, 1961), there is an extensive body of literature that has applied Bayesian modeling successfully not only to describe economic decision-making, but also to capture basic sensorimotor behavior like cue integration, perceptual categorization, visuomotor adaptation, and movement control in both humans and animals (van Beers et al., 1999; Ernst and Banks, 2002; Knill and Pouget, 2004; Körding and Wolpert, 2004; Körding and Wolpert, 2006; Braun et al., 2009).

Nevertheless, Bayesian models have been criticized on several grounds, foremost for their computational intractability, in particular in complex environments, their assumption of perfect rationality, their disregard for model mis-specification as well as their explanatory flexibility when allowing for arbitrary prior beliefs, likelihood models, or cost functions (Jones and Love, 2011). An alternative view to Bayes-optimal decision-making that does not assume an overarching optimization process or principle is sometimes called the Adaptive Toolbox approach to the mind based on many different specialized decision heuristics that are adapted to various environmental contexts (Gigerenzer and Gaissmaier, 2011; Gigerenzer et al., 2011). A key feature of heuristic strategies is that they are fast to apply and that they ignore a large part of the information at hand. Well-known heuristic strategies include the recognition heuristic (Goldstein and Gigerenzer, 2002)—assuming a higher value for an option whose name is recognized—, the take-the-best-heuristic (Gigerenzer and Goldstein, 1996a)—compare two options by their most important criterion and ignore other criteria—and elimination by aspects (Tversky, 1972) that gets rid of alternatives that do not meet the criteria of a certain aspect. Over the years a considerable body of evidence has built up in the literature showing how human decision-makers rely on heuristic decision-making in many real-world scenarios, for example in the financial and medical professions (Julian and Gerd, 2012; Forbes et al., 2015).

Usually, Bayes-optimal decision strategies that integrate all the available information into an optimal decision are regarded as incompatible with the idea of having multiple heuristics that ignore large amounts of information and that are not optimal in any particular sense, even though such heuristic strategies have been shown to work well in practical scenarios where exact models are unavailable or misspecified. Recently, however, there have been several proposals that suggest that heuristics can be regarded as Bayes-optimal strategies under certain constraints. Parpart et al. (2018), for example, have proposed that ignoring certain aspects of data in a heuristic decision-making scheme could be enforced within a Bayesian linear regression framework by putting a high weight on the corresponding prior over regression parameters. In another study (Belousov et al., 2016), Belousov and colleagues have suggested that different reactive and predictive strategies of catching a ball that were previously suggested as heuristic strategies can arise from an optimal stochastic controller when varying the level of observation noise and reaction time. While such adaptive changes in strategy selection depending on task constraints are well-known in the psychological literature (Svenson and Edland, 1987; Payne et al., 1988; Rieskamp and Hoffrage, 2008; Pachur and Bröder, 2013), a general mathematical formalization in terms of a single framework that explains both heuristic and Bayes-optimal decision-making poses an ongoing research problem. Such a framework might not only elucidate the mathematical principles underlying heuristic decision-making, but also shed further light on the question of how heuristics may be learned.

Here, we test an information-theoretic bounded rationality model (Braun et al., 2011; Ortega and Braun, 2011, 2013) for its ability to capture both Bayes-optimal and heuristic decision strategies in human subjects when varying the available information-processing resources in a binary classification task. In this model, limited information-processing capabilities are formalized abstractly by information constraints that quantify how much decision-makers can deviate from a given prior strategy when they face a decision task that is represented abstractly by a utility function. Decision-makers with more resources will be able to deviate more from their priors and adapt to the utility function, whereas decision-makers with limited resources will have to stick more to their prior strategy. The basic rationale is that any kind of resource constraint (e.g., time, memory, money, effort,...) will ultimately translate into a reduced ability for uncertainty reduction (Gottwald and Braun, 2019), which can be considered as an application of the rate-distortion principle of information theory (Cover and Thomas, 2006; Tishby and Polani, 2011). Previously, we have used such information-theoretic bounded rationality models to study effects of resource limitations in motor planning (Schach et al., 2018), absolute identification (Lindig-León et al., 2019), and sensorimotor interactions (Lindig-León et al., 2021).

**Figure 1:**
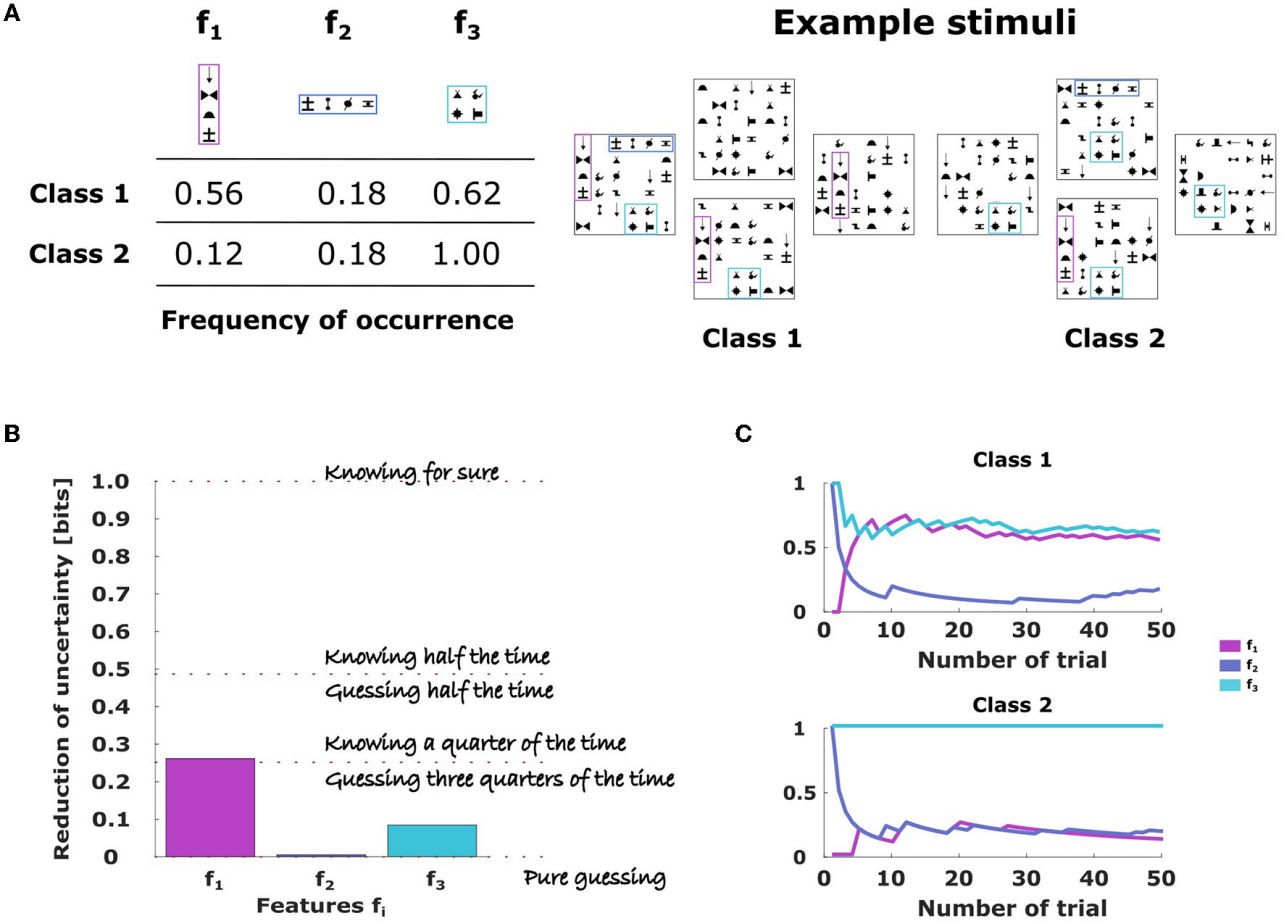
Binary classification task with three features. **(A)** Each feature is given by a symbol arrangement labeled *f*_1_, *f*_2_, and *f*_3_ and occurs with a certain probability for stimuli of each stimulus class, shown in the Table 1. Examples of combined stimulus pattern belonging to class 1 or class 2 are shown on the right. Crucially, subjects did not know the class membership, but had to infer it from the presence and absence of stimulus features. **(B)** The amount of information the different features carry about the stimulus class is measured by the mutual information: a value of 1 bit implies that class membership can be surely inferred, a value of 0 means that the feature provides no information and the decision-maker has to guess. A value in between implies that the feature provides some information and the subject can engage in some educated guessing. **(C)** Temporal evolution of the empirical frequencies of the different stimulus features. Empirical frequencies can be reliably estimated after less than 10 trials.

In our study, we expose human subjects to a binary classification task by two-alternative forced choice, where a stimulus (here: a panel of abstract symbols) has to be assigned to one of two classes based on three different stimulus features (here: certain symbol arrangements)—compare Figures 1A,B. The features have different probabilities for the two stimulus classes and are independent, that means given the class membership the presence of a feature has no information about any other feature. The optimal decision-maker in such a scenario is the Naive Bayes classifier that provides the performance baseline irrespective of the available information-processing resources. This is in contrast to bounded rational decision-makers that optimize performance under given constraints on resources. In particular, we investigate the hypothesis that subjects behave like bounded rational decision-makers that focus on the most informative features under time pressure, thus, ignoring other stimulus information, which corresponds to some kind of Take-the-Best decision heuristic (Gigerenzer and Goldstein, 1996b). To this end, we measure the amount of information subjects extract from the different features and compare that to the information reduction expected by different bounded rationality models—compare. We find that subjects' behavior is best captured by an information-theoretic bounded rationality model that optimizes performance assuming capacity constraints on three separate information channels representing the three features, accounting for the fact that feature information is processed in separate chunks.

## 2. Methods

### Participants

There were a total of 16 participants. They belonged to the age group 20–50, 11 of them conducted the task using remote access. The study was approved by the Ethics Committee of Ulm University. Subjects were informed about the purpose and method of the experiment as well as the ensuing data handling, and that they could withdraw from the study any time. During the initial familiarization phase of the experiment, subjects were explicitly instructed that stimuli were composed of three different features that could occur in isolation or in combination with each other along with distractor symbols. They were shown the three features which were each made up of four abstract symbols inspired by a previous study (Orbán et al., 2008). Subjects were told that they have to classify the stimulus patterns that they will observe into one of two classes and that they can learn this classification from feedback after each trial.

### Experimental design

**Trial design**. In each trial subjects were shown a 6 × 6 square panel (ca. 15 × 15 cm) made of 36 tiles full of blanks and symbols and they were required to press the keys 1 and 2 on the keyboard to indicate whether they believed that the presented stimulus belonged either to class 1 or 2, respectively. The key press had to occur within a given time window depending on the condition (fast condition 1*s*, medium condition 3*s*, slow condition 5*s*, otherwise the trial was counted as a missed trial. After each trial a dialogue box at the center of the screen would indicate to subjects whether the trial was correct, incorrect, or missed. Subjects could have breaks after every block of 50 consecutive trials with the same time constraint (fast, medium, or slow). In total, there were six blocks, that is 300 trials altogether per session, and two sessions per subject. The first three blocks of 50 trials in the first session were executed in the order slow, medium, fast, in the ensuing blocks the order was randomized. Each session lasted approximately half an hour. All evaluations in the paper are based on data from the second session.

**Stimulus classes**. There were two equiprobable classes of stimuli, where each class is defined like a bag-of-words model based on three independent features. In a bag-of-words model such features typically are given by characteristic words whose frequency of occurrence distinguishes, for example, spam from non-spam email. Similarly, in our task, the class-specific occurrence probabilities of specified visual features, each of which could either be present or absent, allows for the classification of stimuli into two separate classes. Based on the three features, we can distinguish eight different stimulus types that can be denoted by a binary vector **x** and that are associated with the two classes with different probabilities. In our experiment we used the following feature probabilities to generate 50 stimuli for each class and condition as a random template:

**Table:** 

**Classes**	**F1**	**F2**	**F3**
**Class 1**	0.616	0.089	0.680
**Class 2**	0.198	0.227	0.985

When subsequently counting the occurrences of features in this template, the empirical frequencies with which features occur did not exactly match the aforementioned probabilities due to the finite amount of stimulus samples. Instead, the empirical frequencies were given by:

**Table:** 

**Classes**	**F1**	**F2**	**F3**
**Class 1**	0.560	0.180	0.620
**Class 2**	0.120	0.180	1.000

We used the same random trial sequence template for all subjects, so we could ensure that each subject experienced exactly the same empirical frequencies. It should also be noted that the empirical frequencies of the trial sequence template could not be known immediately by subjects, but had to be learned. However, as can be seen from Figure 1C, the empirical estimates of the feature frequencies stabilizes already after 10 trials around their final values.

**Stimulus design**. The stimulus design shown in Figure 1 was inspired by a previous study (Orbán et al., 2008). Each stimulus was a 6 × 6 square panel, where each square could contain a symbol or a blank. To balance stimuli in terms of luminosity, each stimulus contained exactly 25 symbols and 11 blanks. The stimuli were characterized by three independent visual features where each feature corresponds to a spatially order chunk of four symbols. The chunks appeared in a fixed part of the panel, but their exact position could vary by two squares at most. Depending on the number of chunks present, more or less randomly placed distractor symbols were used to arrive at the total number of 25 symbols. There was an inventory of 20 different symbols out of which 10 were used to define the three chunks and all of them could be used as distractor symbols.

### Models

The eight possible stimulus pattern can be described by a binary input vector **x** ∈ {0, 1}^3^, for example for the first stimulus pattern with all cues present it would be **x** = (111). The distribution *P*(**x**) over stimulus pattern is given by $P\left(\text{x}\right)=\underset{y\in \left\{1,2\right\}}{\sum }P\left(\text{x}|y\right)\pi \left(y\right)$, where $\pi \left(y\right)=\frac{1}{2}$ is the equal prior probability of presenting a stimulus from either class, and *P*(**x**|*y*) is the generative model of the stimulus pattern in each class *y*. For our task, the generative model is given by


$$\begin{array}{l} P\left(\text{x}|y=1\right)=\overset{3}{\underset{j=1}{\prod }}\mu_{j}^{x_{j}}{\left(1-\mu_{j}\right)}^{1-x_{j}}\\ P\left(\text{x}|y=2\right)=\overset{3}{\underset{j=1}{\prod }}\nu_{j}^{x_{j}}{\left(1-\nu_{j}\right)}^{1-x_{j}} \end{array}$$

where μ_*j*_ and ν_*j*_ are the probabilities of the features occurring in class 1 and class 2, respectively, as determined from the experiment. The decision-maker's choice can be described by a binary output variable *a* ∈ {1, 2} corresponding to a selection of class 1 and class 2, respectively. A choice strategy then corresponds to a conditional distribution *P*(*a*|**x**) detailing the probability of selection class *a* given the stimulus pattern **x**. For example, if **x** = (111), the choice strategy is to assign **x** to class 1 with a probability *p* and to class 2 with probability 1 − *p*.

**Naive bayes model**. The Bayes-posterior for our task is given by the sigmoid $P(y=1|x)=\sigma (w^{T}x+w_{0})$ and *P*(*y* = 2|**x**) = 1 − *P*(*y* = 1|**x**) with the weight parameters


$$\begin{array}{l} w_{j}=\log\frac{\mu_{j}}{1-\mu_{j}}-\log\frac{\nu_{j}}{1-\nu_{j}}\\ w_{0}=\overset{3}{\underset{j=1}{\sum }}\log\frac{1-\mu_{j}}{1-\nu_{j}} \end{array}$$

Assuming a 0/1-utility of *U*(*y, a*) = δ_*y,a*_ with the Kronecker delta δ_*y,a*_ = 1 if *y* = *a* and δ_*y,a*_ = 0 otherwise, we can then define the optimal choice strategy $P\left(a|\text{x}\right)=\delta_{a,a^{*}\left(\text{x}\right)}$ that maximizes the expected utility according to


$$\begin{array}{l} a^{*}\left(\text{x}\right)=\arg\underset{a}{\max}\underset{y}{\sum }P\left(y|\text{x}\right)U\left(y,a\right) \end{array}$$

**Bounded rational model with a single information channel for action**. Assuming the same expected utility as above given by $EU\left(\text{x},a\right)=\underset{y\in \left\{1,2\right\}}{\sum }P\left(y|\text{x}\right)U\left(y,a\right)$, the bounded rational decision-maker with a single information channel for action seeks to find the optimal strategy P^*^(*a*|**x**) that maximizes the expected utility


$$P^{*}\left(a|\text{x}\right)=\arg\underset{P\left(a|\text{x}\right)}{\max}\underset{\text{x}}{\sum }\underset{a}{\sum }P\left(\text{x}\right)P\left(a|\text{x}\right)EU\left(\text{x},a\right)$$

subject to the information constraint


$$I\left(\text{X};A\right)=\underset{\text{x}}{\sum }\underset{a}{\sum }P\left(\text{x}\right)P\left(a|\text{x}\right)\log\frac{P\left(a|\text{x}\right)}{P\left(a\right)}\leq K$$

where the prior is given by $P\left(a\right)=\underset{\text{x}}{\sum }P\left(\text{x}\right)P\left(a|\text{x}\right)$ and *K* is a positive real-valued bound. The optimization problem is equivalent to the unconstrained optimization problem


$$P^{*}\left(a|\text{x}\right)=\arg\underset{P\left(a|\text{x}\right)}{\max}\underset{\text{x}}{\sum }\underset{a}{\sum }P\left(\text{x}\right)P\left(a|\text{x}\right)EU\left(\text{x},a\right)-\frac{1}{\beta }I\left(\text{X};A\right)$$

where the precision parameter β is chosen to match the information bound *K*. The optimal strategy is given by


$$P^{*}\left(a|\text{x}\right)=\frac{1}{Z\left(\text{x}\right)}P^{*}\left(a\right)\exp\left[\beta EU\left(\text{x},a\right)\right]$$

with the optimal prior $P^{*}\left(a\right)=\underset{\text{x}}{\sum }P\left(\text{x}\right)P^{*}\left(a|\text{x}\right)$ and the normalization constant $Z\left(\text{x}\right)=\underset{a}{\sum }P^{*}\left(a\right)\exp\left(\beta EU\left(\text{x},a\right)\right)$.

**Bounded rational model with multiple information channels for action and perception**. Assuming an internal state **s** = (*s*_1_*s*_2_*s*_3_) for perceptual processing and feature-specific information channels *P*(*s*_*j*_|*x*_*j*_) with *j* = 1, 2, 3 that learn to specialize in processing the three different symbol arrangements serving as features, the bounded rational decision problem with multiple information channels for both perception and action can be stated as a joint optimization problem, that does not only seek to optimize the choice strategy *p*(*a*|**s**) given the perceptual information *s*, but also learns to co-optimize the perceptual filters *P*(*s*_*j*_|*x*_*j*_), yielding:


$$\arg\underset{\begin{array}{c} P\left(s_{1}|x_{1}\right)\\ P\left(s_{2}|x_{2}\right)\\ P\left(s_{3}|x_{3}\right)\\ P\left(a|\text{s}\right) \end{array}}{\max}E_{\text{x},\text{s},a}\;EU\left(\text{x},a\right)\;-\frac{1}{\beta }\overset{3}{\underset{j=1}{\sum }}I\left(X_{j};S_{j}\right)-\frac{1}{\beta_{U}}I\left(A;\text{S}\right)$$

where


$$\begin{array}{l} I\left(X_{j};S_{j}\right)=\underset{x_{j}}{\sum }\underset{s_{j}}{\sum }P\left(x_{j}\right)P\left(s_{j}|x_{j}\right)\log\frac{P\left(s_{j}|x_{j}\right)}{P\left(s_{j}\right)}\\ I\left(A;\text{S}\right)=E_{\text{x},\text{s},a}[\log\frac{P\left(a|\text{s}\right)}{P\left(a\right)}] \end{array}$$

with the expectation


$$E_{\text{x},\text{s},a}\left[f\left(\cdot \right)\right]=\underset{x_{1}x_{2}x_{3}}{\sum }\underset{s_{1}s_{2}s_{3}}{\sum }\underset{a}{\sum }P\left(\text{x}\right)P\left(s_{1}|x_{1}\right)P\left(s_{2}|x_{2}\right)P\left(s_{3}|x_{3}\right)P\left(a|\text{s}\right)f\left(\cdot \right)$$

The optimal information channels for perceptual processing of the features are given by


$$\begin{array}{l} P^{*}(s_{1}|x_{1})=\frac{1}{Z(x_{1})}P^{*}(s_{1})\exp(\beta \sum_{x_{2}x_{3}}\sum_{s_{2}s_{3}}P(s_{2}|x_{2})P(s_{3}|x_{3})\\ \;\times P(x_{2}x_{3}|x_{1})F(x,s))\\ P^{*}(s_{2}|x_{2})=\frac{1}{Z(x_{2})}P^{*}(s_{2})\exp(\beta \sum_{x_{1}x_{3}}\sum_{s_{1}s_{3}}P(s_{1}|x_{1})P(s_{3}|x_{3})\\ \;\times P(x_{1}x_{3}|x_{2})F(x,s))\\ P^{*}(s_{3}|x_{3})=\frac{1}{Z(x_{3})}P^{*}(s_{3})\exp(\beta \sum_{x_{1}x_{2}}\sum_{s_{1}s_{2}}P(s_{1}|x_{1})P(s_{2}|x_{2})\\ \;\times P(x_{1}x_{2}|x_{3})F(x,s)) \end{array}$$

with


$$F\left(\text{x},\text{s}\right)=\underset{a}{\sum }P^{*}\left(a|\text{s}\right)EU\left(\text{x},a\right)-\frac{1}{\beta_{U}}\underset{a}{\sum }P^{*}\left(a|\text{s}\right)\log\frac{P^{*}\left(a|\text{s}\right)}{P^{*}\left(a\right)}$$

and the optimal action channel


$$P^{*}\left(a|\text{s}\right)=\frac{1}{Z\left(\text{s}\right)}P^{*}\left(a\right)\exp\left(\beta_{U}\underset{\text{x}}{\sum }P\left(\text{x}|\text{s}\right)EU\left(\text{x},a\right)\right)$$

where


$$P\left(\text{x}|\text{s}\right)=\frac{P\left(\text{x}\right)P^{*}\left(s_{1}|x_{1}\right)P^{*}\left(s_{2}|x_{2}\right)P^{*}\left(s_{3}|x_{3}\right)}{\sum_{x_{1}x_{2}x_{3}}P\left(\text{x}\right)P^{*}\left(s_{1}|x_{1}\right)P^{*}\left(s_{2}|x_{2}\right)P^{*}\left(s_{3}|x_{3}\right)}$$

The overall strategy of the decision-maker is given by


$$P\left(a|\text{x}\right)=\underset{s_{1}s_{2}s_{3}}{\sum }P^{*}\left(s_{1}|x_{1}\right)P^{*}\left(s_{2}|x_{2}\right)P^{*}\left(s_{3}|x_{3}\right)P^{*}\left(a|\text{s}\right)$$

**Bounded rational model by Parpart et al. (2018)**. As an alternative model we consider the bounded rational model by Parpart et al. (2018), that is designed for binary comparisons, where in our case the input **x** ∈ {−1, 0, +1}^3^ indicates whether each cue *x*_*j*_ with *j* ∈ {1, 2, 3} is in favor of class 1 (*x*_*j*_ = +1), in favor of class 2 (*x*_*j*_ = −1) or neither (*x*_*j*_ = 0) and where the output *y* ∈ {−1, +1} indicates whether the stimulus **x** should be assigned to class 1 (*y* = +1) or class 2 (*y* = −1). Due to the feature frequencies in our stimulus set, the presence of the first feature favors class 1, the presence of the second feature is irrelevant, and the presence of the third feature favors class 2. The first stimulus pattern with all cues present would then be expressed as **x** = (+1, 0, −1).

For each feature dimension *j*, the model assumes a different linear mapping between feature vector and outputs


$$y={\text{W}}^{j}\text{x}+W_{0}^{j}$$

where each of the three different weight vectors **W**^*j*^ represents a different linear mapping and $W_{0}^{j}$ is an offset that we added to the model to improve classification performance. The weight vectors are determined by subjects' responses *y*_*i*_ toward stimulus **x**_*i*_ in trial *i* through ridge regression


$$\begin{array}{l} {\hat{\text{W}}}^{j}={\left(\Lambda^{j}+X^{T}X\right)}^{-1}X^{T}\text{y}\\ W_{0}^{j}=\mu_{y}-{\hat{\text{W}}}^{j}\mu_{\text{x}} \end{array}$$

where **y** = (*y*_1_ − μ_*y*_, ..., *y*_*N*_ − μ_*y*_) is the response vector over all trials *N* and $X^{T}=\left({\text{x}}_{1}-\mu_{\text{x}},\ldots ,{\text{x}}_{N}-\mu_{\text{x}}\right)$ is a matrix comprising all stimulus vectors with $\mu_{\text{x}}=\frac{1}{N}\underset{i}{\sum }{\text{x}}_{i}$ and $\mu_{y}=\frac{1}{N}\underset{i}{\sum }y_{i}$, and


$$\Lambda^{1}=\left(\begin{array}{ccc} 0 & 0 & 0\\ 0 & \frac{\sigma^{2}}{\eta^{2}} & 0\\ 0 & 0 & \frac{\sigma^{2}}{\eta^{2}} \end{array}\right)\Lambda^{2}=\left(\begin{array}{ccc} \frac{\sigma^{2}}{\eta^{2}} & 0 & 0\\ 0 & 0 & 0\\ 0 & 0 & \frac{\sigma^{2}}{\eta^{2}} \end{array}\right)\Lambda^{3}=\left(\begin{array}{ccc} \frac{\sigma^{2}}{\eta^{2}} & 0 & 0\\ 0 & \frac{\sigma^{2}}{\eta^{2}} & 0\\ 0 & 0 & 0 \end{array}\right)$$

express the weight that is put on the prior. For η → ∞ the prior is ignored and all three regression weights are identical. For η → 0 each regressor ignores all cue dimensions but one. Each regressor can be seen as a decision hyperplane where $sign\left({\hat{\text{W}}}^{j}\text{x}+W_{0}^{j}\right)$ determines the class membership ±1. To bring the three regressors together for a single action choice, the decision hyperplane is determined which induces the maximum distance


$$j^{*}=\arg\underset{j}{\max}|{\hat{\text{W}}}^{j}\text{x}+W_{0}^{j}|$$

from the stimulus **x**. The class selection is then determined by


$$a=sign\left({\hat{\text{W}}}^{j^{*}}\text{x}+W_{0}^{j^{*}}\right)$$

For η → ∞ this corresponds to a standard regression of the decision hyperplane. For η → 0 each stimulus is classified by the regressor with maximum distance to one of the hyperplanes that ignore all cue dimensions but one, reminiscent of the Take-the-best heuristic.

## 3. Results

**Figure 2:**
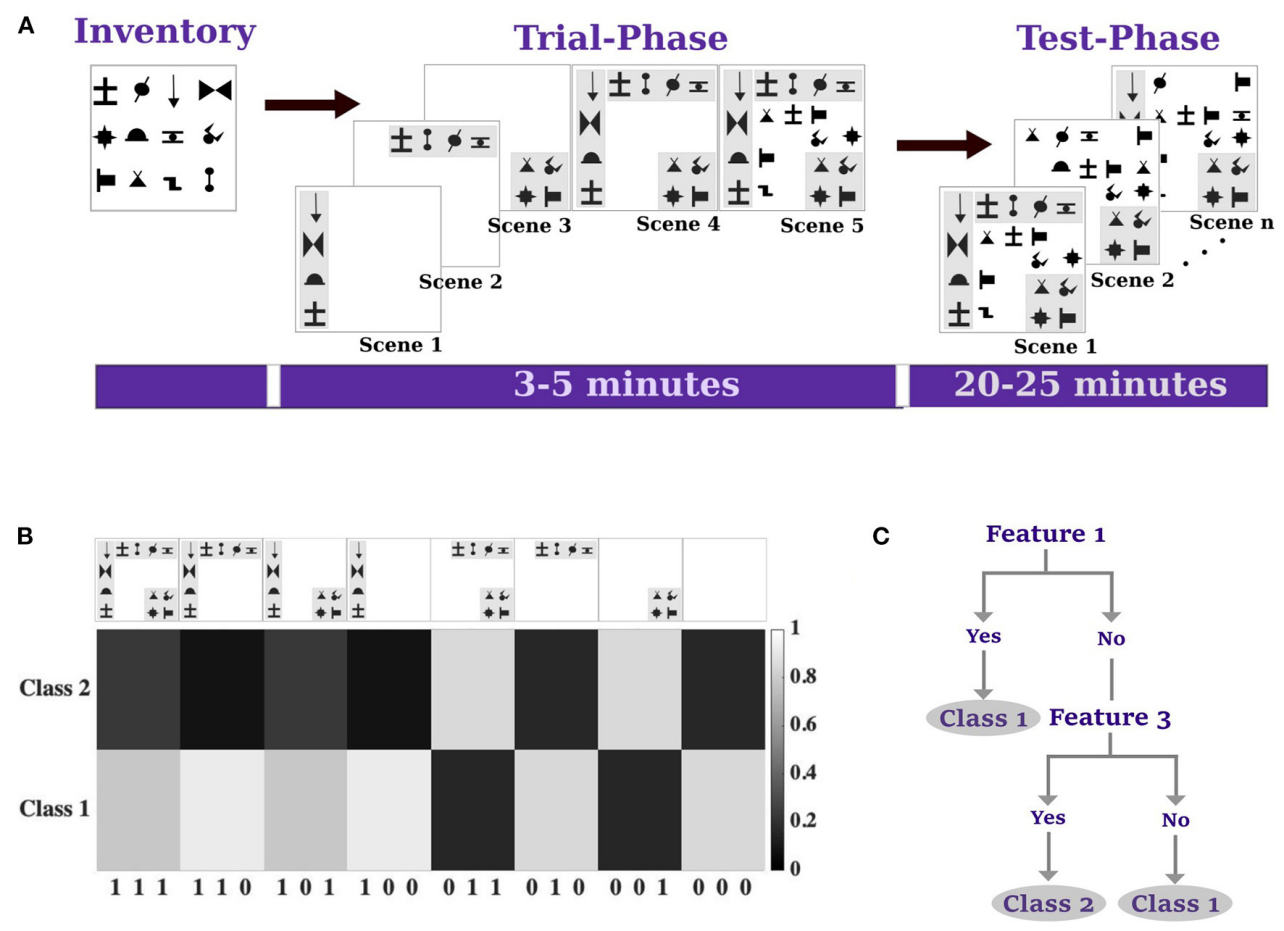
Experimental paradigm and design. **(A)** Inventory of stimulus symbols used. Scenes 1–3 show the three different stimulus features that could be each present or absent in each stimulus. The other symbols were used as distractors. The trial phase was used to familiarize subjects with the stimuli. Example stimuli used in the test phase for learning and testing. **(B)** Eight possible stimulus types indexed by binary triplets to indicate the presence of stimulus features and the associated expected utility given by the probability that the stimulus belongs to Class 1. **(C)** A decision tree that represents the optimal response for the utility function in **(B)**. Under time pressure the decision tree is simplified by replacing the analysis of the third feature by the decision for Class 2.

In the familiarization phase of the experiment, human subjects were first acquainted with the possible symbol inventory that was used to compose stimuli (Orbán et al., 2008). In particular, subjects were shown three different stimulus combinations—shown in Scene 1, Scene 2, and Scene 3 in Figure 2A—that would serve as three independent binary features that could each be present or absent in a stimulus buried among a set of meaningless distractor symbols, as shown examplarily in Scene 5 of Figure 2A. Thus, based on all combinations of binary features, there were eight different kinds of stimuli (disregarding distractors), which we encode in the following by a triplet like 110, meaning feature 1 and 2 are present in the stimulus, while the third feature is absent. A stimulus class is defined by a distribution over the three features. Setting the three features to be independent, this distribution can be defined by three values, e.g., (0.616, 0.089, 0.680) meaning that for stimuli in class 1 the first feature is present in 61.6% of stimuli, the second feature with 8.9%, and so on—compare Figure S1. This way, we can define two different stimulus classes with two different sets of probabilities regarding the presence or absence of the three features and we can determine for each possible kind of stimulus the probability of belonging to class 1 or class 2, respectively—(see Figure 2B). Subjects were then trained on these probabilities by providing them with binary feedback when letting them categorize randomly composed stimuli into the two classes the optimal categorization can be represented by a decision tree - (see Figure 2C). Finally, subjects' performance was tested with the same paradigm after training with three different reaction time conditions ranging from fast, to medium, to slow.

### Behavioral analysis

**Figure 3:**
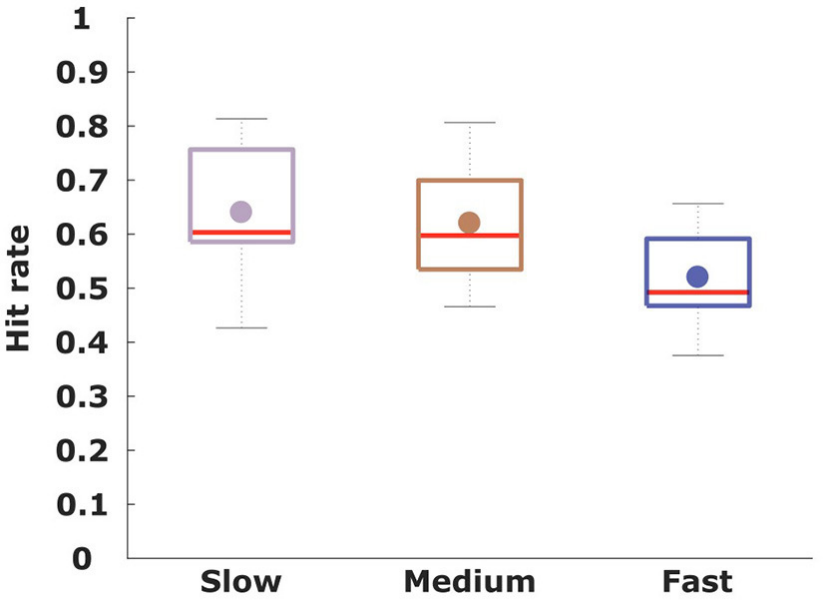
For every condition the hit-rate on the y-axis is averaged across all participants (*n* = 16), indicating how often subjects received positive feedback about their selected stimulus class.

**Figure 4:**
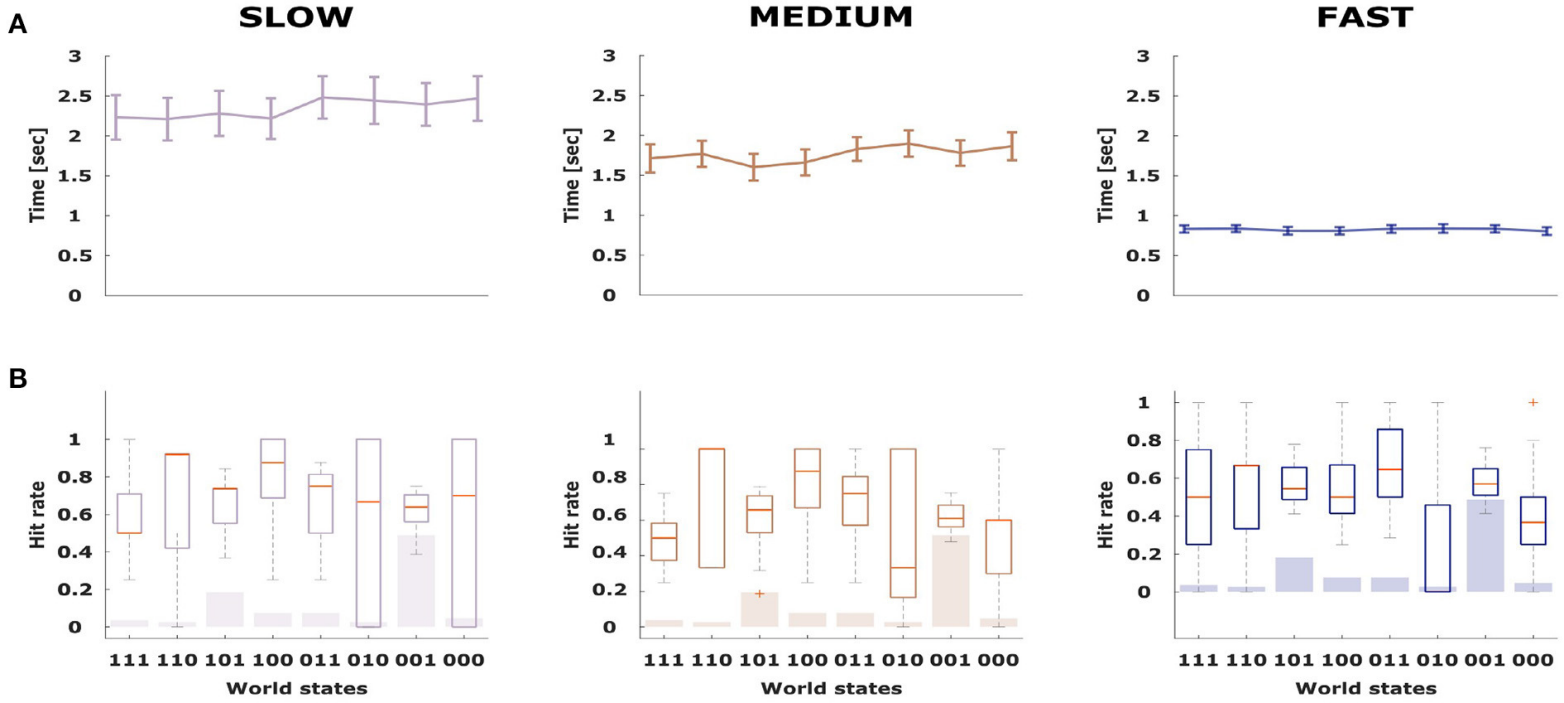
**(A,B)** Average deliberation time for all stimulus types across the three conditions (fast, medium, and slow). Error bars indicate standard error of the mean.

To assess subjects' performance under the three time conditions, we investigated the overall hit rate in each condition averaged across all subjects, reflecting how often subjects experienced positive feedback for their choices. As the stimulus features are only stochastically associated with each class, a perfect hit rate is impossible with a theoretical maximum expectation of 0.80. As shown in Figure 3, the hit rate increased when subjects were given more deliberation time. The performance as measured by the hit rate was 0.64 ± 0.09 in the slow condition, 0.62 ± 0.08 in the medium condition and 0.52 ± 0.07 in the fast condition. Paired *t*-tests revealed significant differences between the mean hit rate of the fast condition compared to the slow (*p* < 0.001, *t* = 4.08, *df* = 15) as well as the medium condition (*p* < 0.001, *t* = 4.08, *df* = 15). The difference in hit rate between the medium and slow condition was not significant (*p* = 0.2, *t* = 1.26, *df* = 15). To investigate whether the hit-rate was particularly increased or decreased for specific stimulus types, we display the stimulus-specific hit rates in Figure 4 overlaid onto the frequency of the different stimuli. The top of the figure shows the corresponding average deliberation time taken by subjects when making their decisions. In the slow condition the deliberation time saturated at around 2.3 s. In the fast and medium condition, the average deliberation time is reduced to around 1.8 and 0.8 s, respectively.

**Figure 5:**
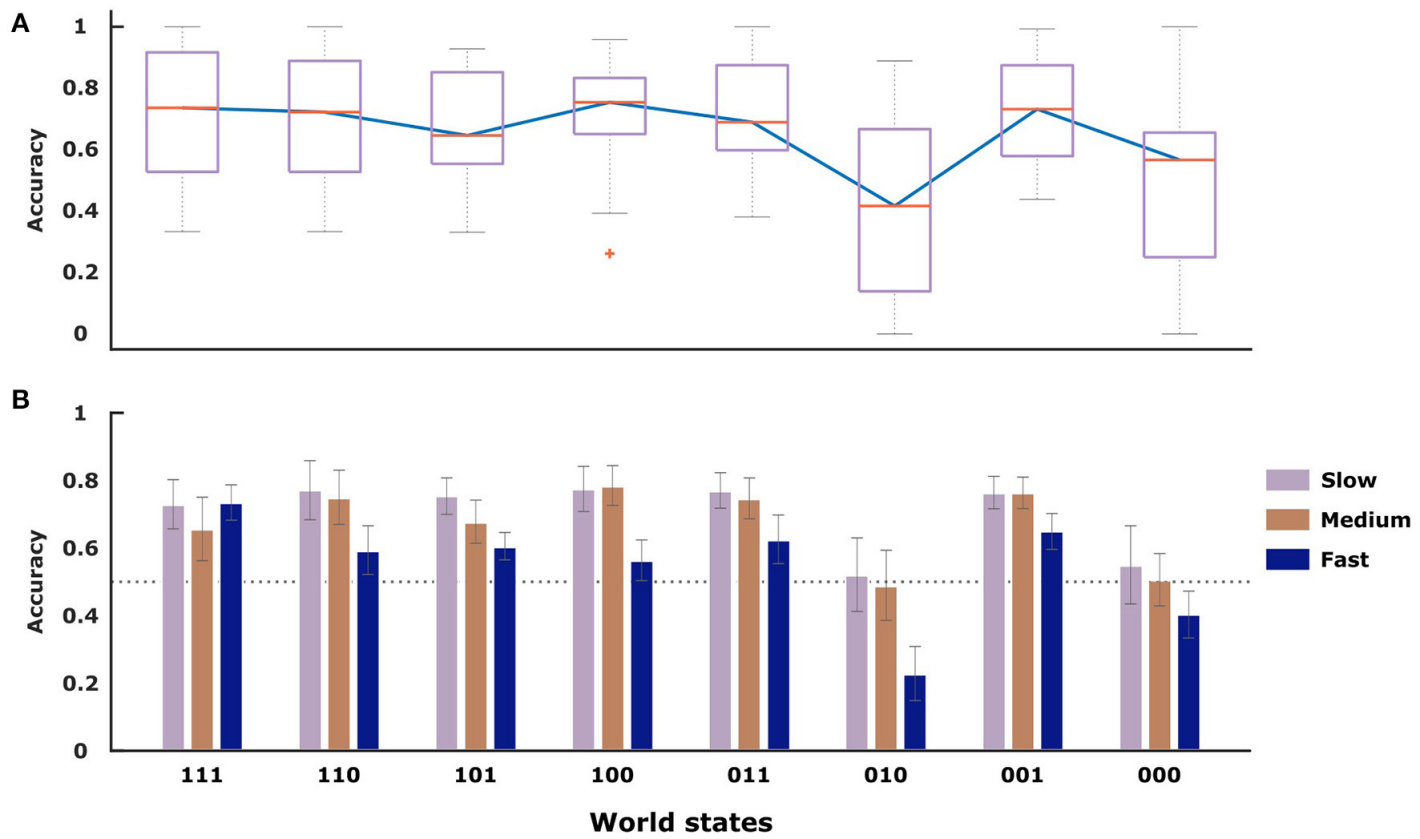
The selection accuracy indicates how frequently subjects chose the class that was more probable. **(A)** Selection accuracy averaged across participants (*n* = 16) for each stimulus type. **(B)** Selection accuracy averaged across participants (*n* = 16) for each stimulus type and condition. The gray error bars show standard error of the mean.

To compare subjects' performance to ideal behavior that always selects the most probable stimulus class in line with Figure 2B, we determined subjects' selection accuracy in Figure 5 for each possible kind of stimulus separately. For an ideal actor it is therefore possible to achieve 100% accuracy (associated with selecting the most probably correct answer all the time), even though the hit rate is only 80% (because of randomness of features). With the exception of the (000)- and (010)-stimulus, all other world states show performance well above the chance line. The median value for the selection accuracy is highest for the (100)-stimulus (0.75 ± 0.05), the (111)- and the (001)-stimulus with values of 0.74 ± 0.05 and 0.73 ± 0.04, respectively. As evident in Figure 5B, with more time there is generally an increase in the mean value of selection accuracy between the three conditions. When performing a repeated measures ANOVA, we find that both factors stimulus (*DF* = 7, *F* = 5.5, *p* < 0.001) and time condition (*DF* = 2, *F* = 12.02, *p* < 0.001) are significant for subjects' selection accuracy. The only exception to this pattern is the (111)-stimulus, despite being associated with a high selection accuracy.

**Figure 6:**
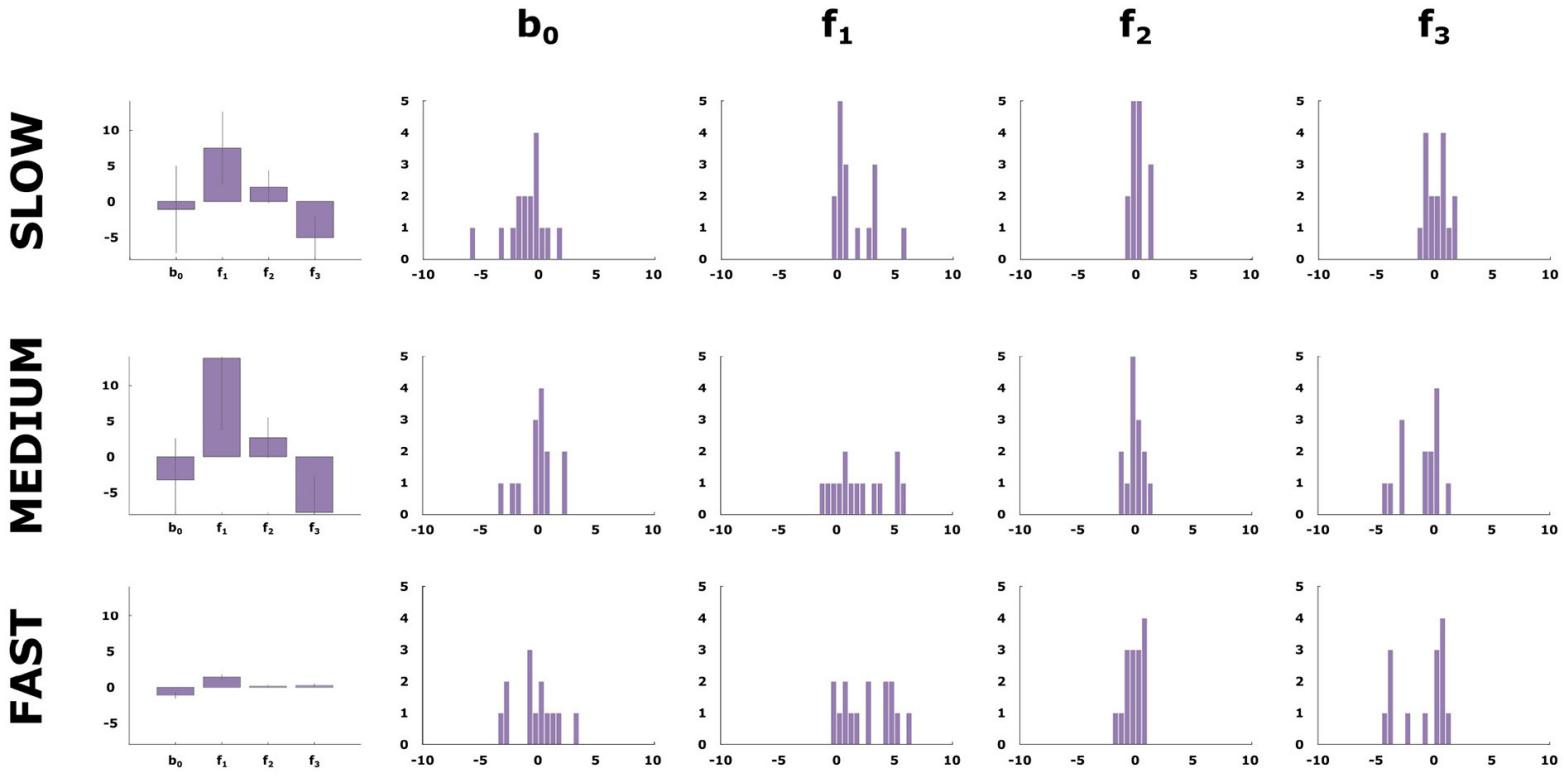
Logistic regression weights. The first column shows the average weight vector values for the logistic regression performed on subjects' selected class in each condition. The next three columns show the distribution of the weight vector values for the 16 participants.

Finally, we assess the relative weight subjects give to the three different stimulus features when making their decisions under the three different time conditions. To this end, we carried out a logistic regression relating subjects' choices to the three different feature dimensions. In Figure 6, it can be clearly seen that there is a considerable dependence on the first feature across all conditions. In contrast, the value of the weight coefficient of the third feature changes significantly from the fast to the slow condition. The presence of this feature corresponds to a higher likelihood for class 2 and it is only with more time resources that the subjects successfully can take this stimulus feature into account. The histogram reveals the distribution of weights across subjects. This shows a consistent strategy change for most subjects with a decision strategy that concentrates on a single feature in the fast condition, something similar to a take-the-best heuristic, and the integration of multiple features in the slow condition.

### Bounded rational modeling

**Figure 7:**
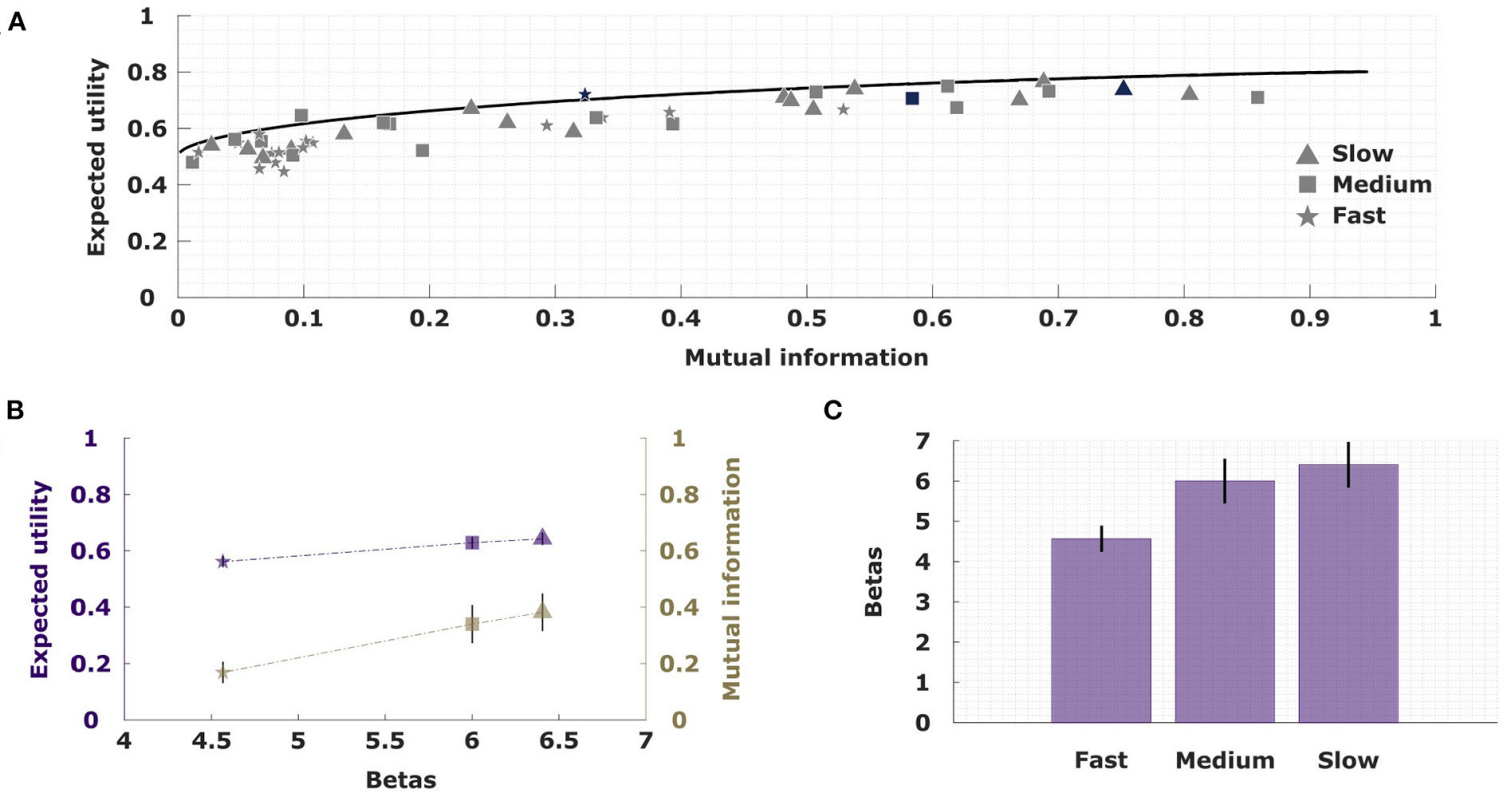
The information-utility trade-off. **(A)** All participants perform close to the optimal information-utility curve (analogous to the rate-distortion curve in information theory). An exemplary participant is highlighted. **(B)** Values of expected utility and mutual information when fitting subjects' behavior with a single information channel model across the three different conditions. **(C)** The precision parameter β that is fitted across the three conditions shows a direct relationship with increasing resources.

Similar to the rate-distortion curve in information theory (Cover and Thomas, 2006), bounded rational decision-making models with a single information constraint allow us to quantify the maximally achievable utility by a decision-maker that has a certain amount of information at their disposal. Here, information measures the extra amount of bits available to encode a posterior class selection strategy that takes into account a particular stimulus pattern compared to a prior class selection strategy that does not know about the stimulus (for example a completely uninformed uniform choice probability, or an optimal prior that is tuned to the statistics of stimulus occurrence). Naturally, the more information-processing capability is available, the higher the maximally achievable utility. This optimal information-utility trade-off is illustrated for our task in Figure 7 assuming a single information channel with an optimally tuned prior. In order to achieve maximum utility with perfection requires a little bit less than one bit of information for binary selection, as the optimal prior strategy is non-uniform due to the non-uniform occurrence probabilities of the stimulus pattern. The non-uniformity of the prior is also evident in the other extreme of zero information, where the expected utility is slightly above the chance level of 0.5.

We can now assess subjects' behavior by measuring the average utility they achieved for each condition and by determining their mutual information between stimulus pattern and class selection based on their empirical choice frequencies. As can be seen in Figure 7A, subjects' performance data lie below the efficiency frontier as expected, but generally close to it. Due to statistical fluctuations a few data points are slightly above. Subjects' data for the fast condition clusters mainly in the low information part of the diagram, whereas performance data of the medium and slow condition are stretched along the efficiency frontier for higher information values. The efficiency frontier is parameterized by a scalar precision parameter, so that we can fit an average precision parameter across all subjects for each condition in a way that minimizes the distance between the data points and the curve. In Figures 7B,C it can be seen that the fitted precision parameter increases across conditions form from fast to slow, and that the average utility achieved by subjects increases together with the average precision parameter.

**Figure 8:**
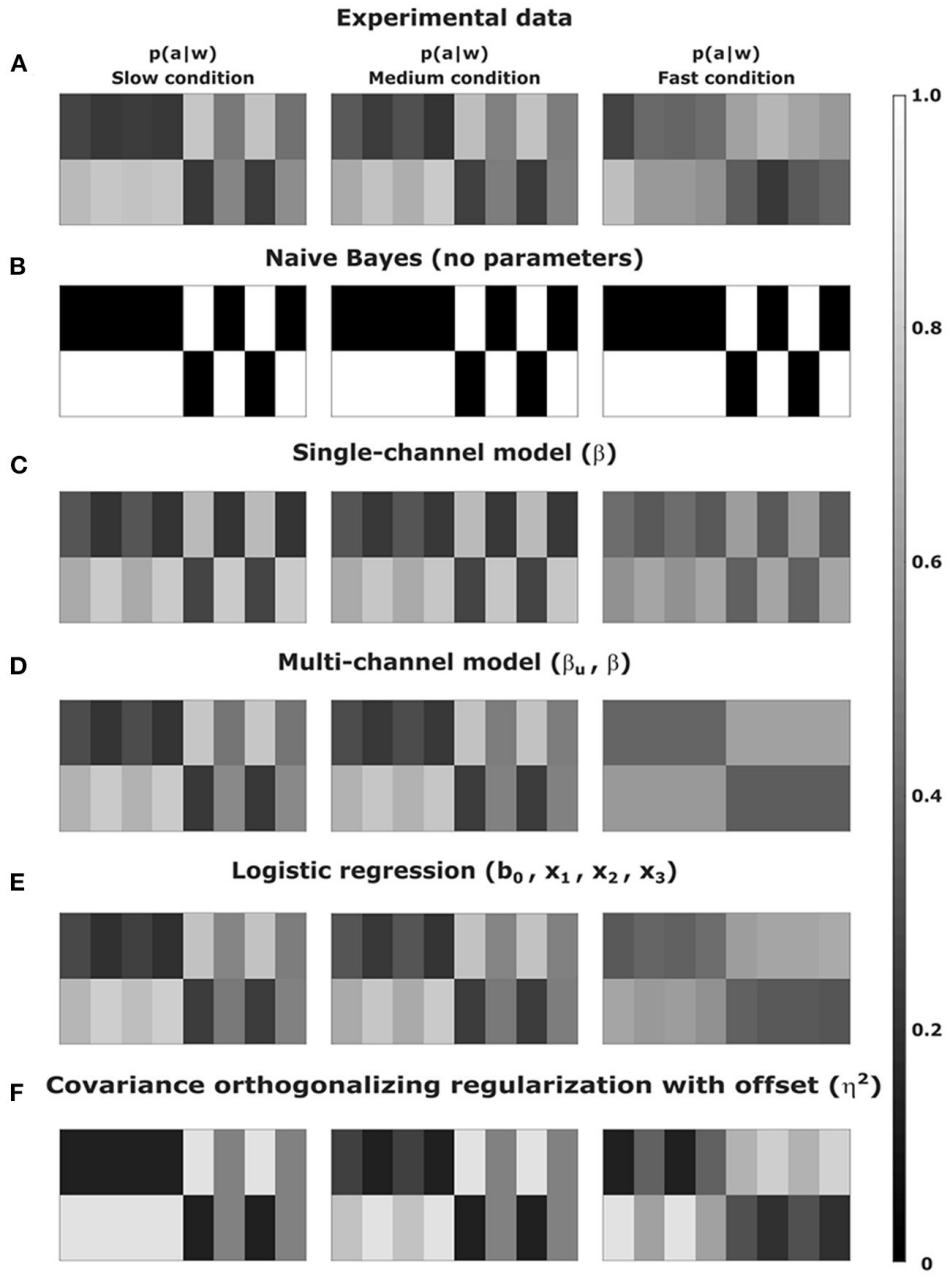
Averaged selection strategies recorded experimentally **(A)** and compared to averaged theoretical selection strategies predicted by a naive Bayes-optimal model **(B)**, a single information channel model **(C)** and a bounded rational model with multiple feature-specific information channels **(D)**, a logistic regression **(E)**, and the bounded rational regression model **(F)** by Parpart et al. (2018).

Depending on the available information-processing resources, the bounded rationality model predicts that the decision-maker's choice strategy should become sharper and more concentrated the higher the available precision is. The choice strategy of the bounded optimal decision-maker more and more optimizes the utility function shown in Figure 2B with increasing time resources. Figure 8C shows the average theoretical posterior of the bounded rational model across subjects fitted by the information constraint determined by the maximum likelihood precision parameter β for each condition and subject. The color code indicates the probability of choosing class 1 or class 2 for each stimulus pattern, and therefore represents a conditional distribution over the class selection variable. Figure 8A shows the average empirical choice strategy formed by subjects across the three time conditions for comparison. The corresponding best responses by the Bayes-optimal model are depicted in Figure 8B and are deterministic and invariant across conditions since they ignore time constraints. We also compare to the average response probabilities of a logistic regression of subjects' responses in Figure 8E where we decrease the number of free parameters across conditions: four free parameters in the slow condition, three free parameters in the medium condition, and two free parameters in the fast condition. Finally, we compare to an alternative regression-based bounded rationality model (COR, Covariance Orthogonalizing Regularization) by Parpart et al. (2018) in Figure 8F. The parameter fits for all models were obtained by maximum likelihood. Table 1 shows the grand average across subjects for each model, the corresponding values for each subject and model for the different time conditions can be found in the Supplementary material in Tables S1–S3.

**Table 1:** Mean model parameters averaged across subjects for the three conditions slow, medium, and fast.

**Model**		**Slow**	**Medium**	**Fast**
sCh	β	1.8823 ± 1.3872	1.7623 ± 1.3214	0.7840 ± 0.7357
mCh	β_*u*_	3.3158 ± 1.5922	3.3026 ± 1.5403	2.7500 ± 1.5655
	β	45.4135 ± 40.2344	45.0724 ± 36.6945	19.2679 ± 16.3503
logReg	*b*_0_	−1.3514 ± 22.3324	−2.9647 ± 21.5730	−1.0700 ± 1.7163
	*x*_0_	7.3524 ± 19.0123	13.3082 ± 37.3361	1.4091 ± 1.7384
	*x*_1_	1.9988 ± 8.5445	2.6053 ± 10.6771	0.1400 ± 0.6096
	*x*_2_	−4.5859 ± 10.2636	−7.4739 ± 18.4158	0.2378 ± 0.9275
COR	η^2^	0.0017 ± 0.0062	0.0046 ± 0.0085	0.0019 ± 0.0060

In the slow condition, the predicted choice probabilities of all models match subjects' data quite well. Compared to the deterministic Bayes-optimal model and the COR model, the bounded rationality model also captures the stochasticity in the choice, including the increase in stochasticity in the medium condition. In the fast condition, the predicted choice probabilities do not match subjects' data so well anymore. As can be seen in the rightmost panel of Figure 8A, subjects' responses tend to fall into two blocks, where one half of the stimulus space is classified predominantly as class 1, and the other half of the stimuli is classified as class 2, mostly. This can be interpreted as a footprint of a decision heuristic that coincides with the simplified decision tree in Figure 2C. However, this bipartite response profile is not reproduced by the naive Bayes model, the COR model or the bounded rational model with a single information constraint, although it is clearly reflected in the logistic regression fit depicted in the rightmost panel of Figure 8E. As suggested by the weights of the logistic regression shown in Figure 6, this change in response profile is due to a change in feature selection.

**Figure 9:**
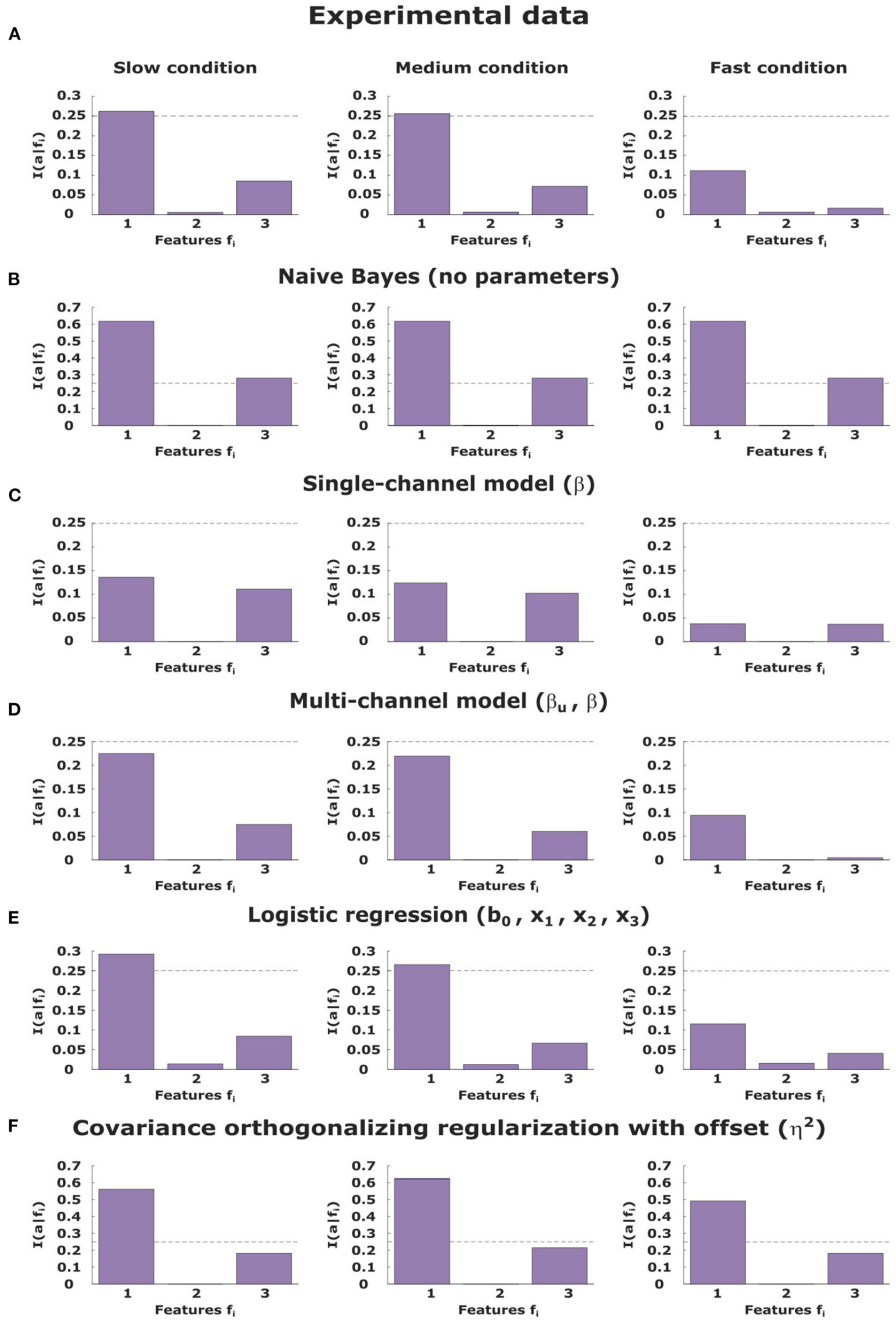
Mutual information between the three stimulus features and the decision-maker's choice as recorded experimentally **(A)** and compared to model fits of a naive Bayes-optimal model **(B)**, a single information channel model **(C)** and a bounded rational model with multiple feature-specific information channels **(D)**, a logistic regression **(E)** and the bounded rational regression model **(F)** by Parpart et al. (2018).

To compare how much weight the different models put on the different feature dimensions of the stimulus with different levels of available information processing capability, we determine in Figure 9 the mutual information between each stimulus feature and the choices subjects made. In line with the logistic regression analysis, we see in Figure 9A that subjects put most weight on the first stimulus feature, largely ignore the second feature, and modulate the importance of the third feature down to the level of the second feature in case of the fast condition with little information resources. Qualitatively, none of the models is able to reproduce this modulation. The naive Bayes model in Figure 9B gives the highest weight to the first feature and a medium weight to the third feature, but the absolute value of the mutual information is too high due to the lack of stochasticity in the response, and more importantly, this profile does not change across conditions, because the model is blind to resource constraints. The COR model in Figure 9F shows a similar response profile that also does not modulate across conditions, because in our experiment there are no correlations between features that the model could downweight. The bounded rational model with information constraint in Figure 9C downregulates the mutual information between actions and features when the task becomes more demanding, however, it does so for all features equally, without showing the observed concentration on the first stimulus features.

The finding that the simple bounded rationality model in Figure 9C fails to capture the transition in the fast condition that is dominated by a single feature may not be too surprising, as the model only allows for a single information channel that cannot distinguish between the different feature dimensions, and therefore lacks internal structure (Luce, 2003). Hence, we also study an extension of the bounded rationality model where we introduce three independent perceptual channels corresponding to the three feature dimensions that are then integrated by the information channel that corresponds to the decision-making process—see section Methods for details. The assumption of a structured internal sensory state with its own precision values allows the model to prioritize between different perceptual states based on their feature grouping and their impact on choice utility, even though *a priori* all feature channels have the same precision. In Figure 8D it can be seen, that, unlike the other models, this extended bounded rational model with feature groupings is able to capture the transition to a bipartite response profile. Moreover, Figure 9D shows that the extended bounded rational model achieves this response profile by downregulating its mutual information between actions and stimulus features the same way that subjects do, namely by focusing almost exclusively on the first stimulus feature.

### Model comparison

To compare the different model fits quantitatively, we take a three-pronged approach: (i) we compare the likelihood of subjects' choices under the probability distributions induced by the various models, (ii) we compare the similarity of the model posteriors to the experimental posterior, (iii) we compare the similarity of the models' mutual information profiles to the experimental profile. The comparison of the mutual information profile is particularly relevant to reveal the models' ability in adapting the feature selectivity across conditions. As a similarity measure we use the Euclidean norm.

To compare the likelihood of subjects' behavior under the different models, we perform a 1, 000-fold cross validation, where in each fold we use a random partition corresponding to half the data for fitting and the other half for evaluation of the likelihood from the predicted probabilities of subjects' choices. The cross-validated likelihoods for each subject and condition are shown in Tables S4–S6. The predicted likelihood of subjects' behavior under the multi-channel model achieves the highest average likelihood of all models. When performing a pairwise comparison, the multi-channel model predicts subjects' choices significantly better than the naive Bayes model (paired *t*-test, *p* < 0.001), the COR model (paired *t*-test, *p* < 0.001), the logistic regression model (paired *t*-test, *p* < 0.001), and the single-channel model (paired *t*-test, *p* = 0.028).

To compare the similarity between the models' posterior and subjects' empirical distribution, we compute the Euclidean norm between the corresponding conditional probability tables and the normalized histograms—compare Tables S7–S9. We find that the multi-channel model has the lowest norm on average. When performing a pairwise comparison, we find that the multi-channel model predicts subjects' conditional choice distribution significantly better than the naive Bayes model (paired *t*-test, *p* < 0.001), the COR model (paired *t*-test, *p* < 0.001), the logistic regression model (paired *t*-test, *p* < 0.001), and the single-channel model (paired *t*-test, *p* = 0.0207).

Finally, we compare how well the models can reproduce the mutual information profile in each condition exhibited by subjects in Figure 9A, by computing the Euclidean distance between the mutual information estimated from empirical choice frequencies and the mutual information profiles computed from model distributions—compare Tables S10–S12. We find that the multi-channel model predicts the best fitting information profile across all conditions compared to any other model (paired *t*-test, pairwise comparison to naive Bayes *p* < 0.001), the COR model (paired *t*-test, *p* < 0.001), the logistic regression model (paired *t*-test, *p* < 0.001), and the single-channel model (paired *t*-test, *p* = 0.0308). More importantly, we find in a separate simulation that merely tries to reproduce the mutual information profile, that the single channel model is unable to generate a profile that focuses on the first stimulus feature in the fast condition, as both the first and the third feature always receive similar weights—compare Figure S2. This suggests that the single-channel bounded rationality model lacks the necessary internal structure to reproduce the adaptive concentration on a single stimulus feature (Luce, 2003), and that a bounded rationality model where perceptual states are grouped according to the presented features provides overall the best explanation of our data.

## 4. Discussion

In this study, we set out to investigate in how far bounded rationality models based on information constraints may provide a bridge between heuristic and Bayes-optimal approaches to decision making in a binary classification task. We collected behavioral data in order to explore the change in strategy that human decision-makers exhibit when taking decisions under varying time pressure. Logistic regression performed on subjects' responses across trials depending on the presented stimulus features reveals a clear pattern for the weight allocated by subjects to each of the features. The dominant weight given to the first feature in the fast condition indicates a focus on a single criterion reminiscent of the take-the-best heuristic (Brusovansky et al., 2018). The broadening of the weights with respect to the other two features, as decision time increases in the medium and slow conditions, suggests that with the introduction of more resources, subjects start to integrate also less informative features to improve decision quality, in line with Bayes-optimal models of information integration (Ernst and Banks, 2002; Körding and Wolpert, 2004). Decision-makers were, thus, not only able to extract and integrate different stimulus features, but were also able to do so in an adaptive manner across conditions (Payne et al., 1988).

The bounded rationality model that we investigate captures the change in strategy for different resource conditions with different information constraints that quantify how much a decision-maker can deviate from their prior choice strategy and how much sensory information they can process from the different stimulus features (Ortega and Braun, 2013). Depending on the information constraints, the model therefore provides a normative standard with which we can compare subjects' performances under different resource conditions (Schach et al., 2018; Lindig-León et al., 2019), as shown in Figure 7. When removing the information processing constraints, the model predictions converge to the deterministic Bayes-optimal decision rule maximizing the expected utility shown in Figure 2B. As predicted by the bounded rational model, over the three time conditions from fast to slow the information divergence measures of subjects' strategy distributions with respect to their prior strategies increase. Furthermore, subjects' average strategies resemble those exhibited by the model under different information constraints in that they become more deterministic in the slow condition, with higher information divergence from the prior, higher utility (lower error rate) and broader weight integration compared to the fast condition. Such a strategy change can of course not be captured by a Bayes-optimal model, like the Naive Bayes model that we evaluated, that does not take into account the available resources.

In the psychology literature, strategy changes under resource limitations have been studied extensively (Payne et al., 1988, 1993), in particular speed accuracy trade-offs that quantify the relationship between task-related error-rates and the time available to perform the task (Heitz, 2014). For example, Rieskamp and Hoffrage (2008) have found in a multi-attribute inference task that increasing time pressure led subjects to a strategy shift from linear additive attribute weighting to a lexicographic heuristic, irrespective of whether time pressure is induced by limiting response times or increasing opportunity costs. A recent study (Oh et al., 2016), very similar to ours, has investigated the strategic shift in a multi-cue classification task with abstract symbol features under varying conditions of time pressure. The task was to choose from a set of two stimuli, the stimulus with maximal winning probability under varying time constraints ranging between 2 s and 700 ms. The winning probability could be determined by integrating over the different symbol features, each of which was associated with a probability of being a winning stimulus. In conditions with little time pressure, the study found that subjects integrated information from multiple features, and that with more time pressure some of the features were ignored, suggesting some kind of cue discounting. The study also found that the observed behavior was not consistent with the take-the-best heuristic, but rather some kind of drop-the-worst heuristic. Such a discounting of less informative cues under time pressure has also been reported recently in a binary classification task modeled with reinforcement learning (Wang and Rehder, 2017) and a recent fMRI study (Oh-Descher et al., 2017) that investigated a shift from cortical to sub-cortical activity with increasing time pressure.

The gradual cue discounting observed under time pressure in previous studies is entirely compatible with our model—compare for example Figure S3 where we apply the multi-channel bounded rationality model to the experiment by Oh et al. with three features (Oh et al., 2016). The model generally predicts that decision-makers should weight the cues depending on their predictive value and that the relative weight of the less predictive cues decreases faster under information constraints. This is consistent with the notion that less informative cues are discounted when time pressure is ramped up. By varying information constraints in a continuous fashion, the bounded rational model is able to describe both gradual transitions in information processing strategies and sharp phase transitions depending on the defined task utilities. Therefore, the prediction of a behavior consistent with a take-the-best heuristic in our task is not intrinsic to the model, but a consequence of applying the model to the particular task. Also in our task, the model does not predict that the less informative features have weight exactly zero, only that the first feature is domineering in the low information regime.

There have been a number of previous studies relating heuristic decision-making rules to utility-based decision models in the context of making choices between gambles. Olschewski and Rieskamp (2021), for example, modeled human decision making under time pressure both with simple heuristics and a classical random utility model. They found that under time pressure decision-makers were more likely to choose risky gambles and to follow more non-compensatory decision strategies. However, their risk model is not applicable to the current task, which is a simple classification task and does not involve the comparison of gambles with different degrees of riskiness. Without risk, the random utility model with a logit link function can be seen as a special case of a single channel bounded rationality model with uniform prior, which does not capture subjects' behavior very well. In another study, Pachur et al. (2017) varied the parameters of a prospect theory model to emulate a number of heuristics for choosing between gambles, including minimax, maximax, and the priority heuristic. Again, our classification task does not involve the comparison of risky gambles, so probability and value distortions cannot reverse the choice between the two classes, only increase or decrease their utility difference, which is the same as a logit model with uniform prior and varying precision parameter. Nevertheless, in more complex decision scenarios the information-theoretic bounded rationality model can also implement minimax and maximax strategies (Ortega and Braun, 2013), while the relation to more elaborate heuristics remains to be explored. Finally, Loewenstein et al. (2015) has investigated the implementation of heuristic choice strategies with accumulator networks, which provides a possible mechanistic explanation for heuristic and bounded rational choices.

Parpart et al. (2018) have previously presented a framework that integrates classical Bayesian rational and boundedly rational decision-making by weighting prior and likelihood in a regression scenario. When the prior is ignored, the resulting logistic regression can take into account correlations between different cues. In the other extreme, each cue dimension is regressed separately and the single most predictive dimension is used for classification as in heuristic decision making. While their model can be applied to our classification task, all cue dimensions in our task are independent, and thus, ignoring cue correlations has no effect. Consequently, the model cannot explain the strategy shifts that we observed under varying time conditions. Previous applications of bounded rationality models with information constraints in tasks with time pressure include sensorimotor tasks like simple reaching movements with varying time pressure for motor planning affecting endpoint precision (Schach et al., 2018), psychophysical tasks like absolute identification of geometrical shapes on different levels of granularity (Lindig-León et al., 2019), and cognitive puzzles like checking satisfiability of conjunctive normal forms with varying deliberation time (Ortega and Stocker, 2016). Compared to these previous studies with information constraints, our current study is the first to apply different information-theoretic bounded rationality models to a multi-attribute decision-making task.

Bounded rationality models come in many different flavors and have a long history going back at least to Herbert Simon's ideas (Simon, 1979) that human decision-makers cannot engage in full cost-benefit analyses when facing complex problems due to their limited cognitive abilities, and that accordingly humans have to exploit heuristics adapted to the structure of their environment in order to find satisfying solutions, that is solutions that are good enough, but not necessarily optimal in any sense. Particularly in psychology, this has led to some researchers completely abandoning the notion of optimality by focusing on process-models of decision-making, that are heuristics used in solving complex practical problems (Gigerenzer and Selten, 2002). While such rules-of-thumb may be outperformed in controlled environments, it has been repeatedly argued that heuristics can perform better than computationally expensive algorithms based on optimality principles, in particular when there is high model uncertainty (Gonzalez, 2004; Buckmann and Şimşek, 2017). However, often the question remains how these heuristics are learned, where they come from and whether there are any underlying mathematical principles. The heuristics-and-biases approach to bounded rationality (Tversky and Kahneman, 1974; Taniguchi et al., 2016; Itri and Patel, 2018), for example, that has focused on the role of cognitive biases in human decision-making has made attempts to formalize biased decision-making procedures using the traditional concepts from economics, however, without normative cogency.

Bounded rationality based on mathematical principles often takes the shape of constrained optimization models in the economics and artificial intelligence literature (Horvitz et al., 1988), where the central idea is that bounded rational behavior can be still regarded as optimal when limitations are taken into account by the appropriate mathematical constraints. Often such models are portrayed as fundamentally irreconcilable with a heuristics approach to bounded rationality, as the remaining notion of optimality apparently introduces an infinite regress: decision-makers that are unable to solve the original utility optimization problem have to solve instead a more complicated optimization problem (Zilberstein, 2008). However, this is only a problem when the bounded rational model is interpreted as a process model, where the decision-maker is optimizing by calculating. When the bounded rationality model only serves as a description for an observer, the model simply allows to compare behavior to a normative baseline. For example, for the bounded rationality model we use in this paper, we could imagine a simple satisficing agent that probabilistically considers random samples with respect to a certain aspiration level only dealing with utility to the best of its abilities, but for an observer it may appear that this decision-making system is trading off utility and information (Ortega and Braun, 2014). Accordingly, the bounded rational model in this paper is still somewhat descriptive and does not provide a detailed heuristic process model, rather it predicts a strategy distribution whose performance is compatible with such a heuristic in our simple task. How this generalizes to more complex tasks is an interesting area for further study.

## Data availability statement

The raw data supporting the conclusions of this article will be made available by the authors, without undue reservation.

## Ethics statement

The studies involving human participants were reviewed and approved by the Ethics Committee of Ulm University. The patients/participants provided their written informed consent to participate in this study.

## Author contributions

NK, CL-L, and DB conceived the experiment. NK conducted the experiment. NK and CL-L analyzed the data. DB developed the model. NK and DB wrote the paper. All authors reviewed the manuscript.

## Funding

This study was funded by European Research Council (ERC-StG-2015–ERC Starting Grant, Project ID: 678082, BRISC: Bounded Rationality in Sensorimotor Coordination).

## Conflict of interest

The authors declare that the research was conducted in the absence of any commercial or financial relationships that could be construed as a potential conflict of interest.

## Publisher's note

All claims expressed in this article are solely those of the authors and do not necessarily represent those of their affiliated organizations, or those of the publisher, the editors and the reviewers. Any product that may be evaluated in this article, or claim that may be made by its manufacturer, is not guaranteed or endorsed by the publisher.
