# Supplementary material for: From Bayes-optimal to heuristic decision-making in a two-alternative forced choice task with an information-theoretic bounded rationality model
Source: Front Neurosci. 2022 Sep 29;16:906198. doi: 10.3389/fnins.2022.906198 (PMC9557085; doi:10.3389/fnins.2022.906198)
Supplement: Supplementary file 1 [file Data_Sheet_1.pdf]

Supplementary Material:
From Bayes-optimal to heuristic decision-making in a two-alternative forced choice task with an information-theoretic bounded rationality model

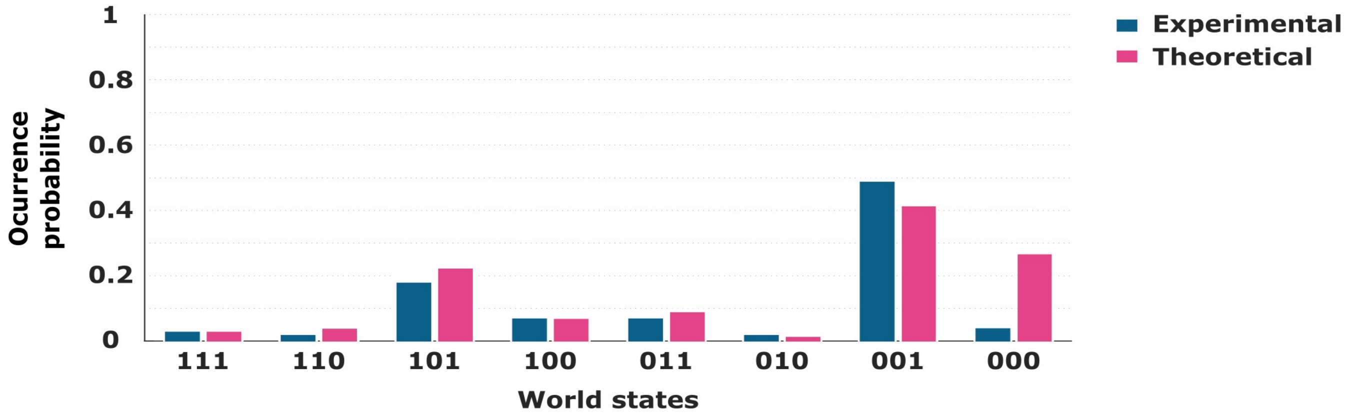

Figure S1: Stimulus frequencies for the eight different stimulus types. Gray bars indicate theoretical probabilities used to generate the random trial template, blue bars indicate actual stimulus frequencies.

		SLOW						
	single Channel	multi channel		logistic regression				COR
Subject	β	β_u	β	b_0	x_0	x_1	x_2	η^2
1	3.62	5.00	32.51	0.42	5.26	-1.67	-3.67	0.0001
2	1.37	2.26	99.75	3.42	0.08	-1.27	-4.04	0.0001
3	4.30	5.00	39.21	0.95	4.95	0.48	-3.6	0.0001
4	2.77	5.00	23.83	-76.27	78.22	33.9	0.94	0.0001
5	2.96	2.89	100.0	32.44	4.3	0.87	-33.78	0.0001
6	3.12	3.11	100.0	24.48	2.64	0.91	-26.64	0.0001
7	2.51	5.00	23.83	-2.52	6.15	-0.93	0.2	0.0001
8	3.28	3.53	51.62	1.69	4.12	1.0	-3.57	0.0001
9	0.12	1.00	100.0	-0.79	1.43	0.23	0.78	0.0005
10	0.29	4.16	3.98	-0.05	0.64	0.02	0.02	0.0248
11	1.82	5.00	9.44	-3.03	4.65	-0.88	1.11	0.0001
12	1.30	5.00	6.71	-2.55	2.96	0.51	0.96	0.0001
13	0.17	1.0	28.29	-0.84	0.7	-0.04	0.79	0.0001
14	0.54	2.05	5.47	-0.5	0.0	-0.11	-0.53	0.0001
15	1.85	1.84	99.75	1.31	1.67	-0.93	-2.43	0.0001
16	0.10	1.21	2.24	0.22	-0.14	-0.10	0.09	0.0001

Table S1. Model parameters for the slow condition.

Subject	single Channel β	MEDIUM multi channel β_u	β	logistic regression b_0	x_0	x_1	x_2	COR η^2
1	3.72	4.58	38.71	0.1	5.11	-0.08	-4.14	0.0001
2	0.89	2.05	87.59	2.03	-1.01	0.83	-2.86	0.0001
3	3.47	5.0	28.79	0.03	34.88	-0.08	-2.58	0.0001
4	2.98	5.0	23.83	-77.78	149.68	42.58	-69.01	0.0001
5	3.31	3.32	100.0	32.26	3.43	1.04	-34.04	0.0001
6	2.30	2.47	99.75	2.39	1.42	-0.34	-3.96	0.0001
7	2.23	5.0	23.83	-3.41	5.61	-0.32	1.19	0.0001
8	3.5	4.58	34.0	0.99	5.14	-0.18	-2.98	0.0001
9	1.02	1.84	56.83	-0.41	1.96	-1.09	-0.16	0.0001
10	0.32	2.89	4.97	-0.02	0.6	0.74	0.03	0.0001
11	1.77	5.0	7.7	-2.22	3.8	-1.2	0.41	0.0001
12	1.3	5.0	5.96	-1.95	2.2	-0.06	0.0	0.0199
13	0.1	2.26	11.67	0.22	-0.59	0.05	0.09	0.0001
14	0.25	1.21	82.38	0.51	-0.21	-0.52	-0.83	0.0099
15	0.96	1.21	99.75	0.05	0.77	0.2	-0.81	0.0226
16	0.1	1.42	15.39	-0.22	0.14	0.11	0.09	0.0206

Table S2. Model parameters for the medium condition.

	FAST							
	single Channel	multi channel		logistic regression				COR
Subject	β	β_u	β	b_0	x_0	x_1	x_2	η^2
1	2.01	5.0	6.71	-1.07	2.85	-0.43	-0.9	0.0001
2	0.1	1.0	41.94	-0.85	0.17	-0.1	0.97	0.0001
3	1.86	3.11	34.5	-0.41	3.08	-0.57	-0.93	0.0001
4	1.96	5.0	7.95	-5.67	5.9	-0.21	1.66	0.0001
5	0.39	1.0	36.73	0.9	0.1	-0.12	-0.62	0.0001
6	0.1	1.63	8.2	-1.72	0.43	0.07	1.46	0.0239
7	1.49	5.0	6.71	-2.44	3.04	0.19	0.52	0.0001
8	1.41	4.16	8.2	-3.2	3.32	1.23	1.64	0.0001
9	0.36	1.21	24.08	-0.42	0.74	-0.1	0.01	0.0001
10	0.1	1.0	59.31	1.71	-0.24	1.06	-1.01	0.0001
11	1.07	3.11	9.44	-1.08	1.57	0.32	-0.01	0.0004
12	0.77	3.74	4.23	-0.77	0.81	-0.64	-0.55	0.0001
13	0.1	1.42	11.17	-0.19	-0.19	0.3	0.47	0.0044
14	0.17	3.53	7.45	-1.83	0.54	1.37	0.94	0.0001
15	0.56	1.0	28.29	0.22	0.41	-0.32	-0.45	0.0001
16	0.1	3.11	13.41	-0.29	0.03	0.16	0.6	0.0010

Table S3. Model parameters for the fast condition.

		SLOW			
Subject	Naive Bayes	Single channel	Multi channel	logistic regression	COR
1	-42.5240	-0.3462	-0.2837	-2.6291	-3.8258
2	-124.357	-0.6194	-0.5883	-11.090	-13.6879
3	-29.3630	-0.2935	-0.2664	-2.7815	-3.6684
4	-65.5378	-0.4388	-0.1564	-0.8625	-3.2209
5	-53.8436	-0.4367	-0.4390	-6.2405	-6.9204
6	-52.9883	-0.3957	-0.4033	-6.4527	-7.0631
7	-73.8164	-0.4899	-0.2366	-2.4546	-4.2862
8	-47.1493	-0.3920	-0.3997	-5.0181	-5.7396
9	-197.748	-0.7012	-0.6992	-16.485	-16.313
10	-187.798	-0.6991	-0.7176	-19.261	-16.416
11	-103.302	-0.5675	-0.3633	-4.4992	-6.4047
12	-131.186	-0.6329	-0.5111	-8.2315	-8.8742
13	-200.310	-0.7020	-0.7185	-19.076	-16.470
14	-189.746	-0.6914	-0.6305	-11.482	-15.424
15	-99.2022	-0.5614	-0.5867	-10.218	-9.9380
16	-202.968	-0.7007	-0.7095	-19.850	-18.323
AVG	-112.615	-0.5418	-0.4819	-9.1645	-9.7861

Table S4. Likelihood of subjects' behavior for the slow condition using a 1000-fold cross validation.

	MEDIUM				
Subject	Naive Bayes	Single channel	Multi channel	logistic regression	COR
1	-41.3976	-0.3365	-0.3280	-3.6147	-4.3918
2	-157.696	-0.6672	-0.5906	-10.134	-16.133
3	-45.0995	-0.3808	-0.2619	-3.3860	-3.7711
4	-60.1076	-0.4247	-0.1294	-0.3831	-2.7134
5	-46.8216	-0.3921	-0.3917	-4.9963	-6.7505
6	-82.1189	-0.4988	-0.5321	-8.8297	-9.7701
7	-84.7277	-0.5212	-0.2763	-2.9608	-4.8150
8	-42.2656	-0.3677	-0.3466	-3.8766	-4.7280
9	-142.779	-0.6597	-0.6331	-11.633	-11.593
10	-182.976	-0.6982	-0.7116	-18.152	-16.543
11	-107.432	-0.5761	-0.4333	-5.4074	-6.8380
12	-140.121	-0.6335	-0.5255	-10.023	-9.8112
13	-241.148	-0.6989	-0.7092	-18.552	-21.112
14	-200.178	-0.6998	-0.7059	-17.084	-17.523
15	-150.747	-0.6628	-0.6917	-14.826	-13.382
16	-209.127	-0.7013	-0.7202	-20.869	-17.848
AVG	-120.921	-0.5575	-0.4992	-9.6517	-10.483

Table S5. Likelihood of subjects' behavior for the medium condition using a 1000-fold cross validation.

	FAST				
Subject	Naive Bayes	Single channel	Multi channel	logistic regression	COR
1	-97.7778	-0.5600	-0.5526	-7.1635	-8.3765
2	-249.295	-0.7000	-0.7227	-20.584	-19.712
3	-98.1674	-0.5698	-0.5095	-6.9982	-8.7339
4	-102.577	-0.5519	-0.3401	-3.3949	-5.2519
5	-179.038	-0.7015	-0.7145	-16.783	-17.865
6	-237.598	-0.6986	-0.7076	-17.084	-18.598
7	-124.312	-0.6149	-0.4907	-6.8044	-8.524
8	-124.947	-0.6246	-0.5384	-7.5467	-8.8158
9	-187.450	-0.6962	-0.7061	-16.931	-15.691
10	-201.552	-0.6999	-0.6186	-10.853	-20.617
11	-144.706	-0.6569	-0.6434	-11.830	-11.783
12	-175.793	-0.6799	-0.6381	-10.674	-13.588
13	-234.471	-0.6984	-0.7089	-19.059	-20.251
14	-214.671	-0.7015	-0.6872	-14.216	-16.263
15	-172.161	-0.6923	-0.7249	-18.828	-15.686
16	-232.096	-0.6984	-0.7056	-18.979	-19.868
AVG	-173.538	-0.6591	-0.6256	-12.983	-14.352

Table S6. Likelihood of subjects' behavior for the fast condition using a 1000-fold cross validation.

			SLOW		
Subject	Naive Bayes	Single channel	Multi channel	logistic regression	COR
1	2.9598	2.5698	2.1009	2.3740	3.1498
2	2.7292	1.4113	0.9484	1.5203	3.8214
3	0.9108	0.9049	0.9203	1.4916	1.8076
4	4.1083	3.8028	0.4939	0.6361	3.2831
5	0.8447	0.7422	0.7628	1.3691	2.0167
6	0.8108	0.5759	0.6297	1.1886	2.0756
7	4.0636	3.6873	0.4318	0.5976	2.6996
8	1.2334	1.0904	1.1813	1.6460	2.1189
9	5.7254	2.1653	1.8924	3.9127	5.0817
10	4.2410	1.6982	1.8518	5.7251	4.4802
11	4.5720	3.5027	0.7665	1.2932	3.6505
12	5.6937	3.5371	1.4270	2.7575	4.2402
13	5.6126	1.8193	1.9361	5.9215	5.5660
14	6.1484	2.1702	1.8292	4.0159	6.1564
15	2.4943	1.3840	1.6185	3.3086	3.0592
16	4.7630	1.9425	2.0287	6.3158	5.6435
AVG	3.5570	2.0628	1.3012	2.7546	3.6781

Table S7. Euclidean norm between the conditional probability and the normalized histogram for the slow condition.

			MEDIUM		
Subject	Naive Bayes	Single channel	Multi channel	logistic regression	COR
1	2.3650	2.0728	2.0020	2.9548	2.5275
2	4.2257	1.8002	1.1119	1.9433	5.0489
3	1.9758	1.9141	1.1871	2.4495	2.1114
4	4.0454	3.8228	0.4738	0.4964	3.0454
5	0.8656	0.7348	0.7390	1.0823	2.2888
6	2.3709	1.2451	1.3189	2.2178	3.6189
7	4.1337	3.6226	0.4655	0.6282	2.8132
8	0.9875	1.0139	1.1121	1.4387	1.9790
9	3.9350	2.1167	1.6947	3.2683	3.5981
10	3.6229	1.6196	1.5820	4.4581	4.0019
11	4.9821	3.5309	1.5445	2.1320	4.0541
12	5.6012	3.1440	1.4721	3.8001	4.3516
13	6.9570	1.9316	2.0448	5.8803	6.8912
14	6.7374	1.9374	1.9618	5.4445	6.6187
15	4.1205	1.7413	2.1501	5.3830	4.5222
16	5.3322	1.4311	1.5795	6.5298	5.2969
AVG	3.8911	2.1049	1.4025	3.1317	3.9230

Table S8. Euclidean norm between the conditional probability and the normalized histogram for the medium condition.

Subject	Naive Bayes	Single channel	Multi channel	logistic regression	COR
			FAST		
1	3.5458	2.5786	2.3692	2.5013	2.2515
2	6.8693	1.8262	1,9237	6.3723	6.4838
3	3.6598	2.8756	1.4835	1.8214	2.9291
4	5.2617	4.0422	1.4132	1.9329	3.8232
5	3.2754	1.8437	1.9281	4.6045	4.3203
6	8.6575	2.2595	2.1196	4.9153	8.1031
7	5.3159	3.3909	1.3952	2.2311	3.5351
8	4.8256	3.1406	1.8449	2.8483	3.6176
9	4.8097	2.0338	1.8578	4.2343	4.2345
10	4.4777	2.5350	1.6315	2.4678	5.3735
11	4.7306	2.2992	1.8952	3.9958	4.1861
12	5.9971	2.6112	2.2403	3.4857	5.3994
13	5.0828	1.7527	1.8639	6.3185	5.1000
14	6.9619	2.0465	2.0532	4.9285	6.4531
15	5.0458	1.8413	2.1475	6.4641	5.3706
16	6.1712	1.5070	1.6316	5.9783	5.9206
AVG	5.2930	2.4118	1.8624	4.0688	4.8189

Table S9. Euclidean norm between the conditional probability and the normalized histogram for the fast condition.

Subject	Naive Bayes	Single channel	Multi channel	logistic regression	COR
			SLOW		
1	0.2121	0.2536	0.1535	0.3049	0.3316
2	0.6036	0.1657	0.1098	0.4900	0.6810
3	0.2014	0.1961	0.1556	0.2732	0.3095
4	0.3073	0.6003	0.2766	0.1582	0.2874
5	0.3049	0.1496	0.1533	0.3167	0.3819
6	0.4172	0.1649	0.1698	0.4535	0.5179
7	0.2616	0.4812	0.1395	0.2911	0.2872
8	0.2762	0.1702	0.1736	0.3057	0.3702
9	0.6172	0.0636	0.0644	0.4680	0.6858
10	0.6513	0.0333	0.0415	0.4648	0.7201
11	0.3198	0.3694	0.1409	0.4402	0.3766
12	0.4543	0.2126	0.1321	0.5651	0.5428
13	0.6523	0.0324	0.0426	0.5092	0.7032
14	0.6632	0.0309	0.0376	0.1226	0.7396
15	0.5155	0.1074	0.1319	0.4692	0.5939
16	0.6650	0.0174	0.0189	0.4350	0.7423
AVG	0.4451	0.1905	0.1213	0.3792	0.5169

Table S10. Euclidean norm between the mutual information estimated from empirical choice frequencies and the mutual information profiles computed from the models for the slow condition.

	MEDIUM				
Subject	Naive Bayes	Single channel	Multi channel	logistic regression	COR
1	0.1997	0.2307	0.1935	0.3784	0.4172
2	0.6244	0.1418	0.1083	0.5495	0.6744
3	0.1953	0.3596	0.1721	0.3084	0.2741
4	0.3354	0.6285	0.3334	0.1259	0.2958
5	0.3233	0.1498	0.1504	0.3611	0.4437
6	0.5324	0.1718	0.1806	0.4942	0.6185
7	0.2769	0.4619	0.1310	0.3525	0.3160
8	0.2022	0.2556	0.1831	0.2808	0.3304
9	0.5512	0.1228	0.1020	0.7466	0.6192
10	0.6534	0.0431	0.0487	0.3503	0.7182
11	0.3758	0.2784	0.1446	0.5473	0.4453
12	0.5292	0.1337	0.0947	0.5647	0.6434
13	0.6526	0.0312	0.0313	0.6378	0.7230
14	0.6610	0.0327	0.0417	0.4608	0.7012
15	0.6248	0.0642	0.0834	0.6748	0.6956
16	0.6655	0.0174	0.0192	0.4982	0.7403
AVG	0.4627	0.1952	0.1261	0.4582	0.5410

Table S11. Euclidean norm between the mutual information estimated from empirical choice frequencies and the mutual information profiles computed from the models for the medium condition.

	FAST				
Subject	Naive Bayes	Single channel	Multi channel	logistic regression	COR
1	0.1819	0.2298	0.2066	0.6909	0.6504
2	0.6528	0.0492	0.0482	0.4686	0.6910
3	0.3695	0.2471	0.1744	0.6332	0.5259
4	0.3073	0.3936	0.1822	0.4227	0.3759
5	0.6561	0.0418	0.0439	0.2213	0.7386
6	0.6507	0.0535	0.0558	0.3229	0.6492
7	0.4281	0.2327	0.1537	0.6647	0.5782
8	0.4569	0.2135	0.1453	0.6924	0.6286
9	0.6445	0.0396	0.0503	0.7325	0.7475
10	0.6587	0.0531	0.0537	0.0640	0.7222
11	0.5798	0.0981	0.0957	0.7735	0.6907
12	0.6348	0.0578	0.0890	0.1877	0.7342
13	0.6613	0.0261	0.0261	0.5043	0.7745
14	0.6608	0.0583	0.0660	0.2343	0.7460
15	0.6536	0.0408	0.0527	0.6329	0.7389
16	0.6631	0.0246	0.0244	0.3630	0.7234
AVG	0.5662	0.1162	0.0918	0.4756	0.6697

Table S12. Euclidean norm between the mutual information estimated from empirical choice frequencies and the mutual information profiles computed from the models for the fast condition .

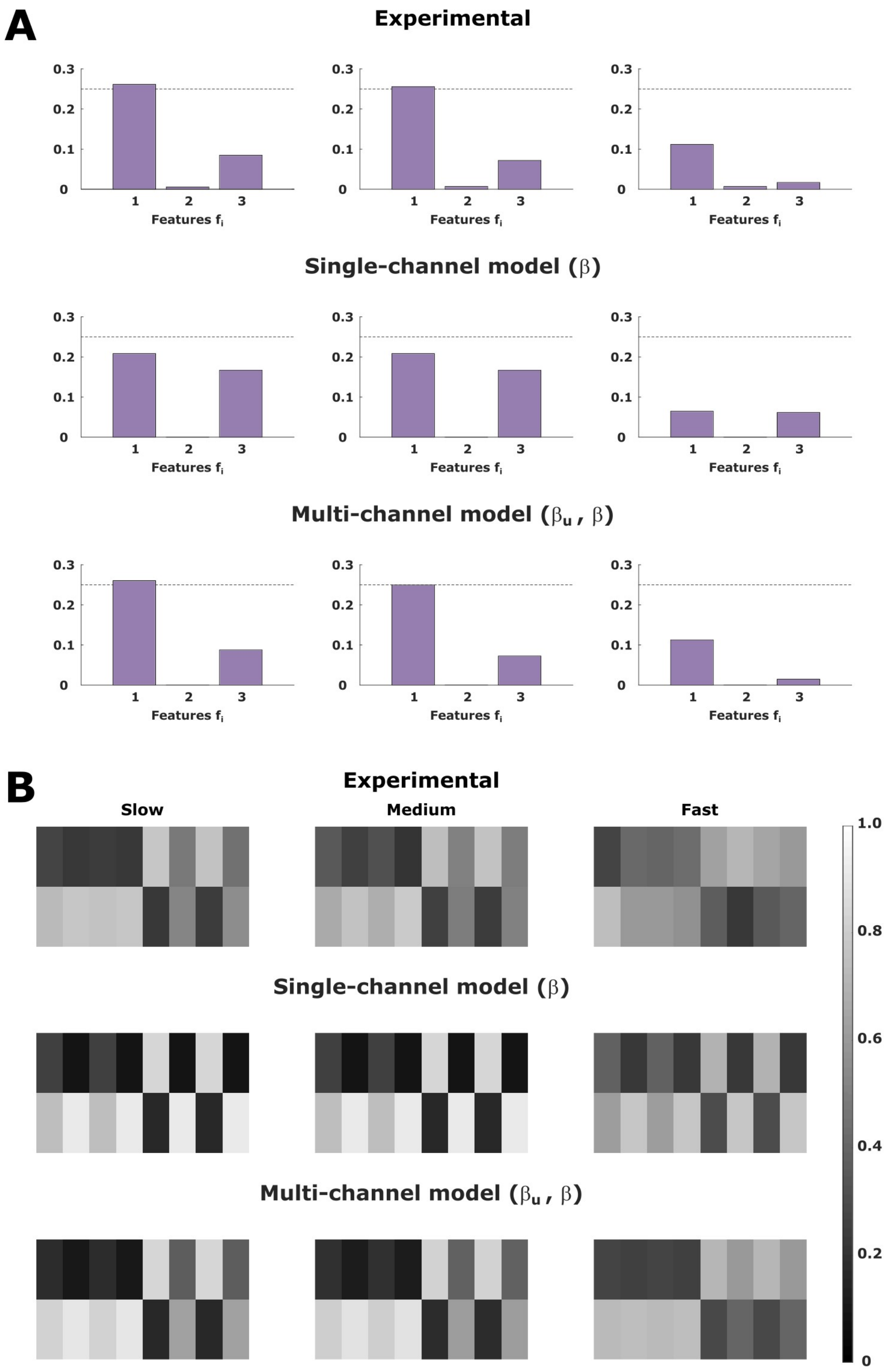

Figure S2: Fit of mutual information. We fit the parameters of the single- and multi-channel model to match the average experimental mutual information profile as precisely as possible by minimizing the Euclidean norm of the deviation in mutual information. The best-fitting parameters are $\beta = 2.7$ and $\beta = 42.93/\beta_U = 3.11$ for the single and multi-channel model respectively in the slow condition, $\beta = 2.7$ and $\beta = 44.42/\beta_U = 2.89$ for the single and multi-channel model respectively in the medium condition, and $\beta = 1.32$ and $\beta = 58.32/\beta_U = 1.84$ for the single and multi-channel model respectively in the fast condition. (A) Unlike the multi-channel model, the single channel model is unable to reproduce a mutual information profile in the fast condition that focuses almost exclusively on the first feature. (B) The posteriors associated with the mutual information fits also confirm that the predicted choice pattern of the single-channel model do not match the experimentally observed average choice pattern that essentially bipartitions the stimulus space.

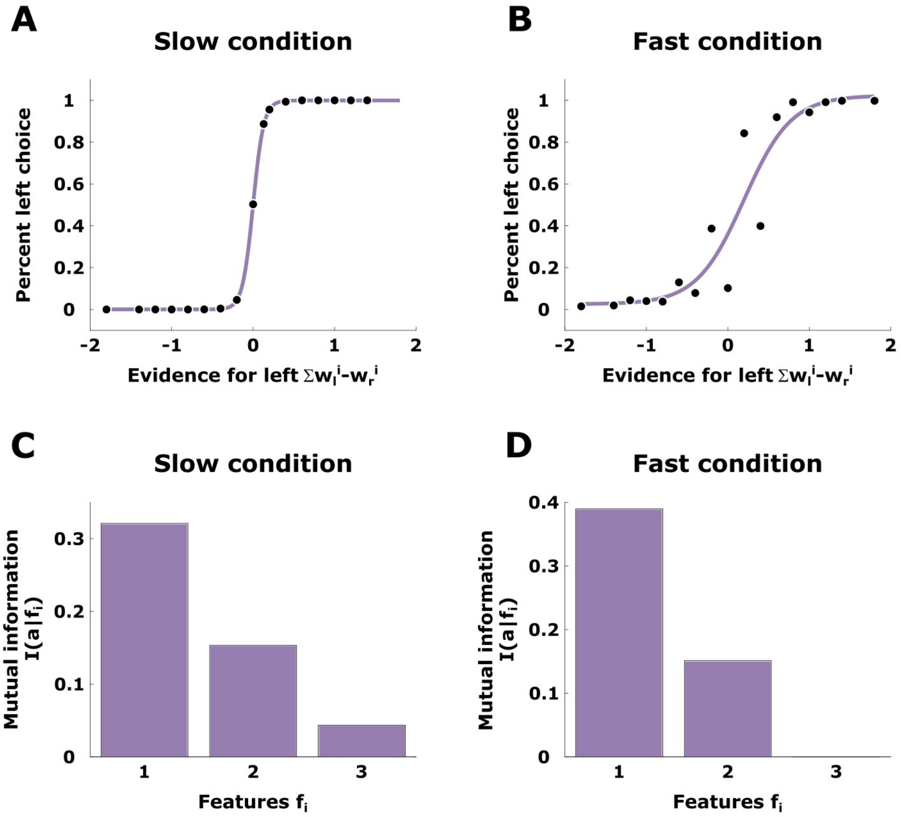

Figure S3: When applying our model to the task of Oh et al. [1] with three feature channels, we need to specify stimuli, prior and utility. Each stimulus $\mathbf{x}$ consists of two options with three binary features each, weighted by $w_1 = 0.9$, $w_2 = 0.8$ and $w_3 = 0.7$ to mark the winning option. Thus, in total there are 64 stimuli. The prior is given by $p(\mathbf{x}) = \frac{1}{64}$ and the utility is given by

$$\begin{aligned} U(\mathbf{x}, a = op1) &= \frac{10^{\sum_{i=1}^{3} w^i_{op1} - w^i_{op2}}}{1+10^{\sum_{i=1}^{3} w^i_{op1} - w^i_{op2}}} \\ U(\mathbf{x}, a = op2) &= 1 - U(\mathbf{x}, a = op2). \end{aligned}$$

In our simulation of the slow condition ($\beta = 80, \gamma = 20$) all features are taken into account and the psychometric curve is steeper. In our simulation of the fast condition ($\beta = 20, \gamma = 10$) only the two most informative features are taken into account and the psychometric curve is shallower due to increased variability.

REFERENCES

[1]Hanna Oh, Jeffrey M. Beck, Pingping Zhu, Marc A. Sommer, Silvia Ferrari, and Tobias Egner. Satisficing in split-second decision making is characterized by strategic cue discounting. *Journal of Experimental Psychology: Learning, Memory, and Cognition*, 42(12):1937–1956, 2016.
